# Mechanical Resonant Sensing of Spin Texture Dynamics in a 2D Antiferromagnet

**DOI:** 10.1002/adma.202420168

**Published:** 2025-04-30

**Authors:** S M Enamul Hoque Yousuf, Yunong Wang, Shreyas Ramachandran, John Koptur‐Palenchar, Chiara Tarantini, Li Xiang, Stephen McGill, Dmitry Smirnov, Elton J. G. Santos, Philip X.‐L. Feng, Xiao‐Xiao Zhang

**Affiliations:** ^1^ Department of Electrical & Computer Engineering University of Florida Gainesville FL 32611 USA; ^2^ Institute for Condensed Matter Physics and Complex Systems School of Physics and Astronomy The University of Edinburgh Edinburgh EH9 3FD UK; ^3^ Department of Physics University of Florida Gainesville FL 32611 USA; ^4^ National High Magnetic Field Laboratory Tallahassee FL 32312 USA; ^5^ Donostia International Physics Centre (DIPC) Donostia‐San Sebastian 20018 Spain; ^6^ Higgs Centre for Theoretical Physics The University of Edinburgh Edinburgh EH9 3FD UK

**Keywords:** 2D materials, magnetic properties, NEMS, spintronics

## Abstract

The coupling between the spin degrees of freedom and macroscopic mechanical motions, including striction, shearing, and rotation, has attracted wide interest with applications in actuation, transduction, and information processing. Experiments so far have established the mechanical responses to the long‐range ordered or isolated single spin states. However, it remains elusive whether mechanical motions can couple to a different type of magnetic structure, the non‐collinear spin textures, which exhibit nanoscale spatial variations of spin (domain walls, skyrmions, *etc*.) and are promising candidates to realize high‐speed computing devices. Here, collective spin texture dynamics is detected with nanoelectromechanical resonators fabricated from 2D antiferromagnetic (AFM) MnPS_3_ with 10^−9^ strain sensitivity. By examining radio frequency mechanical oscillations under magnetic fields, new magnetic transitions are identified with sharp dips in resonant frequency. They are attributed to collective AFM domain wall motions as supported by the analytical modeling of magnetostriction and large‐scale spin‐dynamics simulations. Additionally, an abnormally large modulation in the mechanical nonlinearity at the transition field infers a fluid‐like response due to ultrafast domain motion. The work establishes a strong coupling between spin texture and mechanical dynamics, laying the foundation for electromechanical manipulation of spin texture and developing quantum hybrid devices.

## Graphical abstract

Detection of antiferromagnetic spin texture in a 2D magnetic crystal is achieved through nanomechanical resonators at radio frequencies. Sharp magnetic transitions that lead to abrupt changes in mechanical linear and nonlinear responses are assigned to antiferromagnetic domain motions. The results indicate rich and fluid‐like dynamics between the coupled spin and lattice at the transition field.

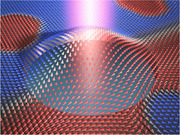

## 1 Introduction

In most conventionally studied magnetic materials, the spin states establish long‐range orderings and, therefore, can be simplified to a single or several spin sublattices, enabling them to be easily addressed by micro‐ and macroscopic approaches.^[^ ^1^, ^2^, ^3^, ^4^, ^5^, ^8^ ^]^ In contrast, spin textures display nanoscale spatial variations of complex spin orientations and host a variety of exotic magnetic structures, including domain walls (DWs),^[^ ^10^, ^13^ ^]^ skyrmions,^[^ ^11^ ^]^ helical magnets,^[^ ^14^ ^]^ and spin liquids.^[^ ^15^ ^]^ Spin texture excitations in antiferromagnets (AFMs), where the spin states have compensated magnetic moments, are particularly interesting and offer new perspectives in designing information processing and logic devices.^[^ ^9^, ^10^, ^11^, ^12^, ^16^, ^17^, ^18^, ^19^ ^]^ The strong magnetic exchange interactions in AFM can lead to rich spin configurations and fast dynamics in the GHz to THz range.^[^ ^20^, ^21^ ^]^ However, detecting and manipulating these complex nanoscale spin textures and dynamics are particularly challenging due to the vanishing overall magnetic moments.

Recent advances in van der Waals (vdW) magnetic crystals offer new opportunities to investigate and manipulate spin interactions in low‐dimensional systems.^[^ ^22^, ^23^, ^24^, ^25^ ^]^ Exotic spin textures can be found and on‐demand engineered in various two‐dimentional (2D) magnets and magnetic heterostructures like moiré structures^[^ ^26^, ^27^, ^28^, ^29^ ^]^ with potential electrical tunability.^[^ ^24^ ^]^ In particular, the robust mechanical stabilities and outstanding elastic properties of 2D vdW materials provide unique advantages for designing efficient nanomechanical devices and enable new device geometries that cannot be realized in 3D or conventional thin‐film magnetic systems. Here, we report the mechanical sensing of nanoscale spin texture formation in 2D AFM materials enabled by exchange magnetostriction. Magnetostriction, which is the lattice change due to spin interactions, has applications in both classical^[^ ^5^ ^]^ and quantum transduction and sensing.^[^ ^6^, ^7^ ^]^ Magnetometry based on such magneto‐mechanical effects has been applied in single‐spin and long‐range ordered (ferromagnetic and AFM) magnetic systems. Examples include the sensing of a single electron spin,^[^ ^8^, ^30^ ^]^ ferromagnetic stochastic processes,^[^ ^31^ ^]^ and magnetic phase transitions^[^ ^32^, ^33^ ^]^ with magnetized mechanical cantilevers and membranes. In the formation of spin textures, the rearrangement of neighboring spins leads to changes in magnetic exchange interactions, especially in AFM materials with large exchange energies. The corresponding exchange‐induced attraction or repulsion of neighboring sites thus yields a new equilibrium lattice position and macroscopic mechanical changes to minimize the system's total energy. While AFM spin textures and domains are conventionally difficult to detect due to vanishing magnetization and nanoscale sizes, we expect high‐sensitivity magnetostriction measurements to enable characterizations of the spin texture formation and dynamics.

**Figure 1:**
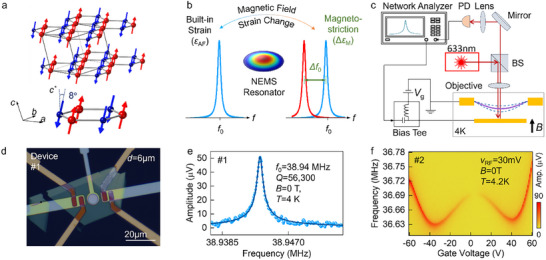
MnPS_3_ nanoelectromechanical resonator devices. a) The crystal structure of few‐layer MnPS_3_. The spin orientation of Mn atoms is represented by the up and down arrows. b) Conceptual illustration of magnetostriction induced resonance frequency shift due to the spin‐flop transition in MnPS_3_ mechanical resonators. Due to magnetostriction effects, the strain changes in the membrane leadto shifts in the resonance frequency. c) Optical interferometry system to measure the driven response of the resonators. BS: beam splitter, PD: photodetector. The 4 K cryostat has a superconducting magnet that can sweep the magnetic field in both directions. The applied magnetic field is along theout of plane direction of the suspended membrane. d) The microscopy image of a MnPS_3_ nanoelectromechanical resonator (device #1, d = 6 µm). Scale bar: 20 µm. e) Measured resonance in MnPS_3_ resonator (device #1). Fitting to a finite‐Q damped harmonic resonator model exhibits resonance at f_0_ = 38.94 MHz with Q = 56300 at 4 K. f) Resonance frequency tuning by varying the DC gate voltage (device #2). Resonance frequency tuning demonstrates a W‐shaped tuning curve with a wider |V_G_| sweep range.

To achieve ultrasensitive mechanical sensing of the spin texture, we fabricated 2D AFM material MnPS_3_ into nanoelectromechanical systems (NEMS) operating in the radio frequency (RF) range. MnPS_3_ is a 2D Heisenberg‐type van der Waals AFM below its Neel temperature of 78 K.^[^ ^34^, ^35^, ^36^, ^37^ ^]^ **Figure** **1**a illustrates the corresponding spin structures. At zero magnetic field, the spins are fully compensated within each of the layers and collinearly aligned with a tilting angle of ≈8° relative to the perpendicular direction.^[^ ^37^ ^]^ Under an out‐of‐plane magnetic field, a gradual spin‐flop (SF) transition is expected near 4–5 T.^[^ ^34^, ^35^ ^]^ The magnetization of bulk MnPS_3_ was measured and included in Section S2 Supporting Information). The MnPS_3_ NEMS resonators with low dissipation (therefore, a high quality factor *Q*) enable a high‐sensitivity detection of magnetostriction effects under a varying field, as depicted in Figure 1b. The magnetic field dependence of the linear and nonlinear dynamics of the 2D AFM mechanical resonators exhibits two sharp magnetic transitions, one close to the expected SF field, and the other being a completely new transition at a higher field ≈7 T. Analytical modeling and atomistic spin‐dynamic simulations indicate that these sharp transitions arise from fast domain wall (DW) motions at the switching fields. The large modulation in macroscopic mechanical properties is attributed to the coupling of rapid local spin texture dynamics and mechanical resonant motions. Furthermore, the abnormal sign change of third‐order (Duffing) and enhancement of fifth‐order (quintic) mechanical nonlinearity terms suggest a fluid‐like DW motion and dynamic process near the SF field. This work establishes a new detection scheme of nanoscale spin textures and opens up a pathway to manipulating novel spin excitations with engineerable mechanical resonance modes.

## 2 Results and Discussion

### 2.1 Mechanical Resonator Device Characterizations

Mechanically exfoliated MnPS_3_ flakes were transferred and suspended over circular cavities with varying diameters prefabricated on a sapphire substrate to form the NEMS structures with controlled mechanical degrees of freedom (see Experimental Section). Figure 1d shows the white‐light microscopy image of a typical device. The suspended 2D MnPS_3_ membrane forms a drumhead resonator with a fundamental flexural‐mode resonance frequency determined by the geometry, Young's modulus, and strain (see Section S4, Supporting Information). An RF voltage combined with a DC gate voltage is applied to the local back gate to electrically drive the membrane to vibrate, and the membrane oscillatory displacement can be detected through laser interferometry (Figure 1c). The measurement system details can be found in Experimental Section. In this report, we focus on the mechanical fundamental resonance mode measurements. Figure 1e shows the resonance of a 25 nm‐thick MnPS_3_ drumhead, exhibiting a very high *Q* factor of ≈56000 at 4 K (device #1). When applying a DC back gate voltage, we can electrically tune the material's strain with the correspondingly exerted static Coulomb force. A DC gate voltage sweeping up to ±60 V yielded a *W‐*shaped frequency tuning curve, which demonstrates the interplay between capacitive softening and elastic stiffening (Figure 1f). Combined with the extracted Young's modulus (see Sections S8–S13, Supporting Information, and Experimental Section) of this material, we estimate a lattice parameter change resolution down to the attometer (10^−18^ *m*) level, which corresponds to $\frac{\delta a}{a}\approx 10^{-9}$ (*a*: lattice constant) and membrane transverse displacement sensitivity of femtometer scale, enabled by such a high *Q* factor.

### 2.2 Magnetic Field Dependence of Linear and Nonlinear Resonator Responses

**Figure 2:**
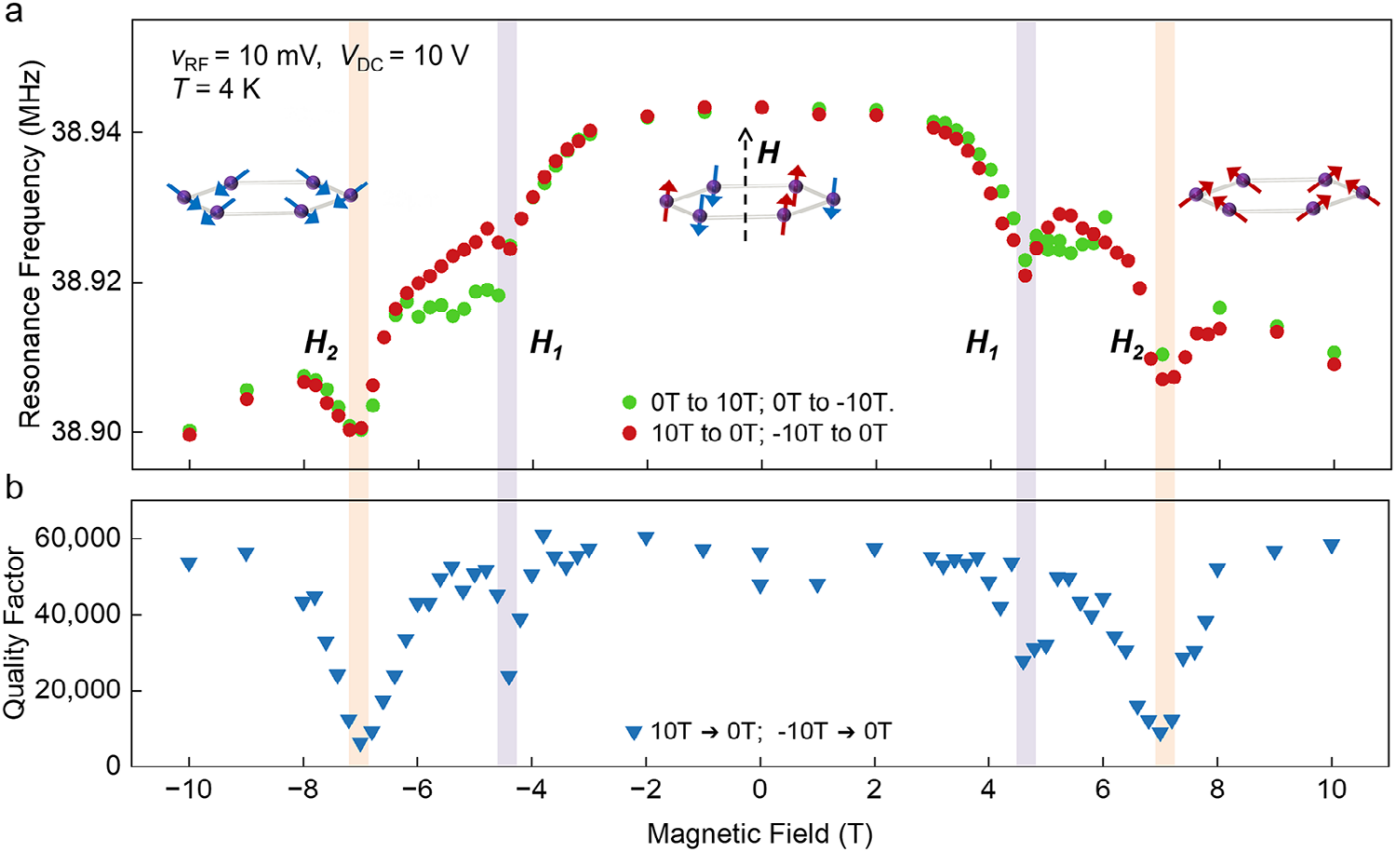
Magnetic field dependence of mechanical resonator in linear region. a) Evolution of resonance frequency as a function of out‐of‐plane magnetic field measured between ±10 T. A complete cycle is achieved by sequentially adjusting the magnetic field μ_0_H from 0 T to 10 T, then decreased from 10 T to −10 T, and finally returned to 0 T. Sharp transitions are observed near μ_0_H_1_ = ±4.6 T and μ_0_H_2_ = ±7 T, as indicated by the purple and orange stripes. The insets plot the corresponding spin configurations: AFM below the SF transition (middle) and canted‐spin states above the SF transition (left and right). b) Measured Q factor versus magnetic field shows strong dissipation at the sharp transitions.

An out‐of‐plane magnetic field was applied while measuring the mechanical resonance. We first examined the magnetic field dependence with the resonator operating in its linear regime with a perturbative small RF driving voltage (10 mV). **Figure** **2**a shows the resonance frequency shift while sweeping the magnetic fields. In the range of −3T and +3T, the resonator frequency remained roughly constant. With further increasing field, the frequency showed an overall decrease. Most notably, two sharp frequency drops were observed at ±4.6 T (*H*_1_) and at ±7 T (*H*_2_), which are direct indications of sudden tension reduction and mark the presence of two distinct magnetic transitions. In comparison, when the device was warmed up above the Neel temperature (100 K, see Figure S13, Supporting Information), no sharp dip‐ or shoulder‐like transitions were observed, confirming the magnetic origin of these two dips. The *H*_1_ transition is close to the SF transition that was previously observed in neutron scattering and magnetic susceptibility measurements.^[^ ^34^, ^36^, ^38^ ^]^ The spin configuration is expected to transit from the AFM to canted states across the SF transition, as depicted in the Figure 2a insets. On the other hand, the *H*_2_ transition has not been identified in direct magnetization characterization. While the SF transition is expected to be first‐order in nature and possesses a hysteresis field, the theoretically calculated hysteresis gap is less than 0.02 T and thus does not contribute to the *H*_2_ transition.^[^ ^39^ ^]^ A previous magnetoresistance measurement on exfoliated MnPS_3_ also reported a slope change in the tunneling magnetoresistance at ≈7 T, which was at that time assigned to be the gradual rotation of SF.^[^ ^36^ ^]^ The abrupt frequency dip observed at *H*_2_ transition here indicates a different origin.

The two transitions show distinctly different hysteretic responses. The *H*_1_ transition exhibits a sharper dip with a decreasing magnetic field sweeping, while the increasing field sweeping gives more shoulder‐like gradual transitions. The *H*_2_ transition, on the other hand, has no hysteresis and possesses a broader transition width compared to *H*_1_. The extracted transition fields for devices of several different MnPS_3_ thicknesses are plotted in Figure S14 (Supporting Information) and summarized in Table S1 (Supporting Information). Our results show that there is a slight decrease in the transition fields with decreasing thicknesses, which is consistent with prior reports of layer‐dependence in MnPS_3_^[^ ^36^ ^]^ and indicates a decrease in magnetic interactions with the reducing layer number.

Accompanied by the frequency dips, the *Q* factor decreases up to one order of magnitude at these two transition fields. The inverse of the *Q* factor (*Q* ^−1^) reflects the damping in the mechanical resonance mode, and our observations indicate the opening‐up of strong dissipation channels at *H*_1_ and *H*_2_. At the measurement temperature (4 K), dephasing is not expected to contribute significantly to the Lorentzian spectral width,^[^ ^40^ ^]^ and the *Q* factor is directly related to the ring‐down time τ of the resonator by *Q*  =  π*f*_0_τ, where *f*_0_ is the peak frequency. We can then estimate the time‐constant τ_*M*_ of the new dissipation channel by considering a *Q* factor drop from 6 × 10^4^ to 6 × 10^3^ at *f*_0_ ≈ 39 MHz, which gives τ_*M*_ ≈  50 µs at the *H*_2_ transition.

**Figure 3:**
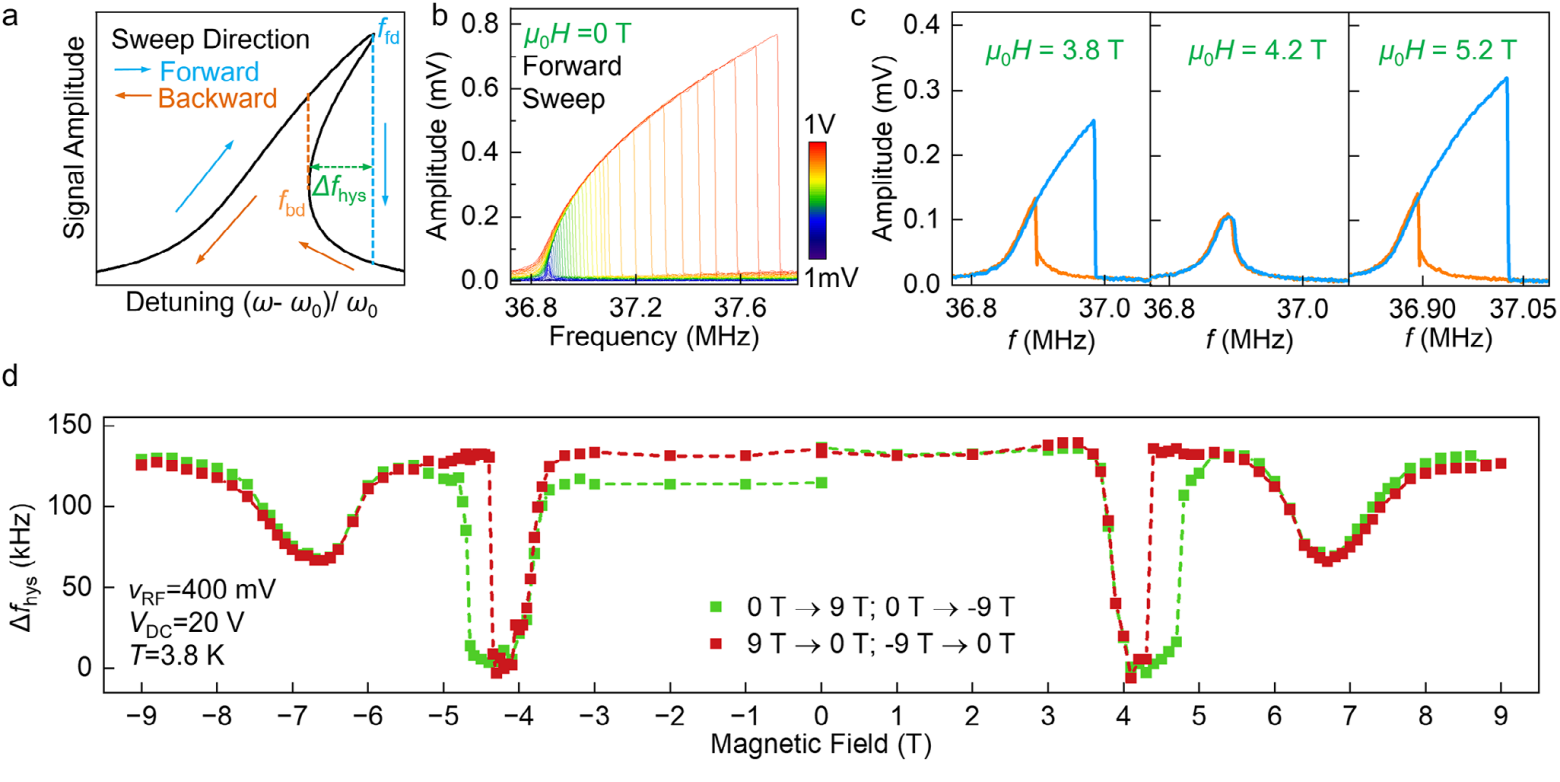
Magnetic field dependence of Duffing nonlinearity. a) Conceptual illustration of Duffing nonlinearity with increased drive force. Forward and backward sweeps show a frequency hysteresis due to Saddle Node bifurcation. b) Duffing nonlinearity measurement by increasing v_RF_ from 1 mV to 1 V at zero field. c) Evolution of Duffing nonlinearity in device #2 at varying magnetic field. At 4.2 T, the forward and backward sweeps coincide with each other. d) Measured frequency hysteresis (Δf_hys_) during forward and backward frequency sweeps versus out‐of‐plane magnetic field shows characteristic sharp dips at the transition fields. The transition at 4.2 T exhibits hysteresis under increasing and decreasing magnetic fields.

**Figure 4:**
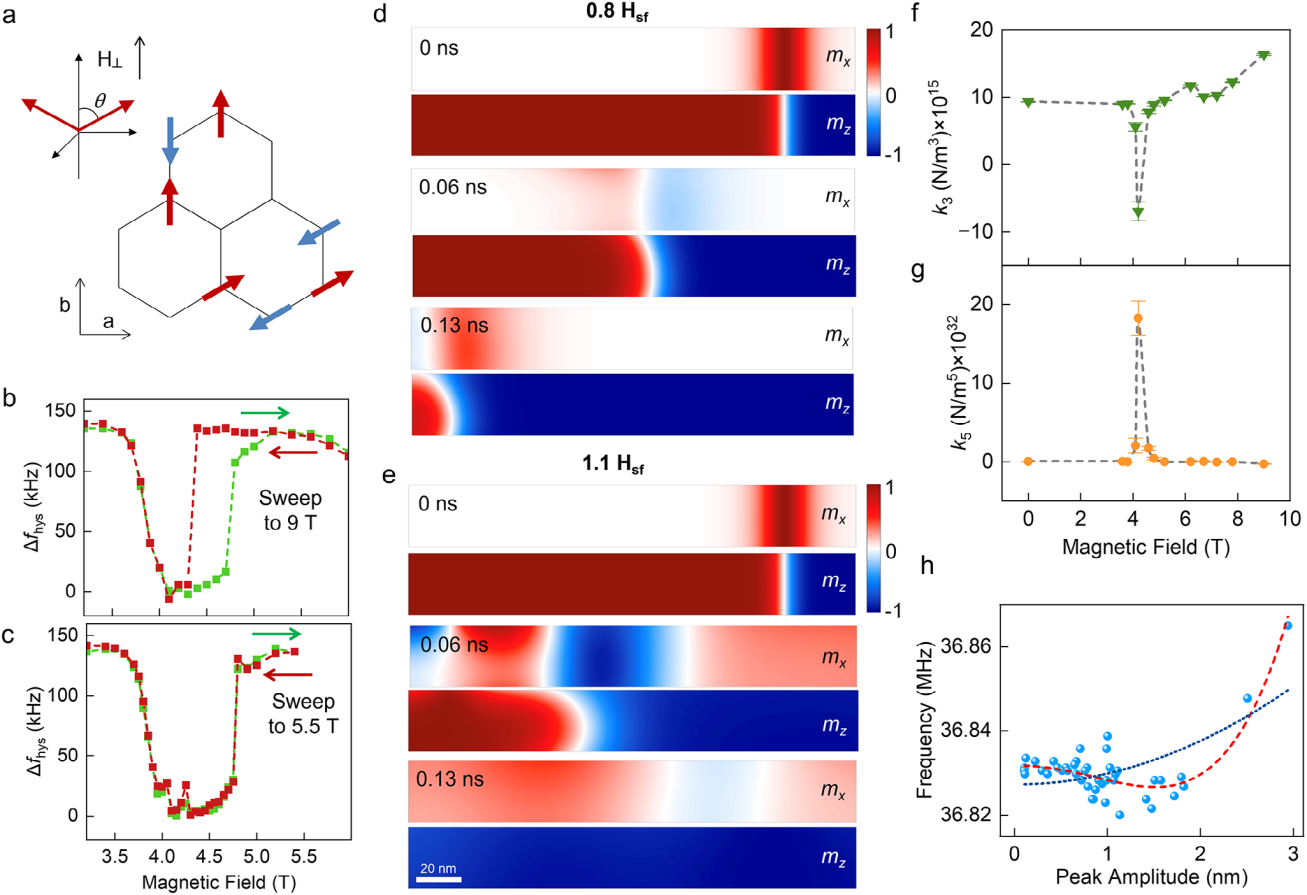
Complex magnetic domains in magnetic transition. a) Possible in‐plane spin orientations above the spin‐flop field. Due to large exchange and small anisotropy, the canting angle in a spin‐flop state, as plotted in the upper right corner, is close to 90° under a moderate out‐of‐plane magnetic field. b,c) Comparison of the different hysteresis of nonlinear signals. The hysteresis is missing when the upper field sweep stops below *H*_2_. d,e) Spin dynamics simulations for AFM domains at magnetic fields of 0.8 H_sf_ and 1.1 H_sf_, respectively, of the spin‐flop field (H_sf_). Panels showthe projected *m_x_* and *m_z_* components of the AFM magnetization at different time‐steps (0, 0.06, and 0.13 ns). A domain‐wall is initially stabilized at the system with an odd number of layers (5L) and its dynamics is followed at 4.2 K. f,g) The magnetic field dependence of *k*_3_ (in **f**) and *k_5_* (in **g**) from the backbone curve. h) The extracted backbone curve at *μ*_0_ *H*_1_ = 4.2 T is shown. The red dashed line is the fitting that includes *k*_5_ while the blue dashed line is the fitting that includes only up to *k*_3_.

We further examine the results when driving the MnPS_3_ resonator at high RF power levels that induce Duffing nonlinear resonator behavior.^[^ ^41^ ^]^ The restoring force of a mechanical resonator under a small displacement *z* is written as $F\;\left(z\right)=F_{0}+kz+k_{3}z^{3}+k_{5}z^{5}+\cdots$. When the higher‐order terms of *k*_3_ (and in fewer cases, of *k*_5_) are included under a strong driving condition, the resonance peak becomes asymmetric with bifurcation hysteretic jumps between the forward and backward frequency sweeps, as depicted in **Figure** **3**a. Figure 3b displays the evolution of linear to nonlinear Duffing response as a function of the increasing RF drive voltage in device #2. Here, we use the frequency gap Δ*f*_hys_ between the hysteretic jumps to characterize the magnetic field dependence of Duffing nonlinearity. Figure 3d plots the Δ*f*_hys_ of MnPS_3_ NEMS resonator (device #2) with RF driving voltage of 400 mV. The two sharp transitions of *H*_1_ and *H*_2_ are clearly manifested in the nonlinear response. The prominent hysteretic behavior at *H*_1_ is also consistent with the hysteresis in linear resonator frequency shifts in Figure 2a. As shown in Figure 3c, at the *H*_1_ of 4.2 T, the resonance peaks completely lost the hysteretic frequency jumps compared to other fields outside the transition points. Such modification in nonlinearity is particularly abrupt for the downward field sweep across *H*_1_. The nonlinear spring coefficients are analyzed in detail in Section S9 (Supporting Information) using models ignoring the damping terms. The corresponding results are summarized in **Figure** **4**f,g, which will be discussed later.

To understand the nature of the *H*_1_ and *H*_2_, we first estimate the expected magnetostriction effects based on the SF transition and previously measured magnetic interactions in MnPS_3_. We model the AFM spin structure and the SF transition as coupled two‐spin sublattices with an easy axis along the out‐of‐plane direction, which is simplified from the slightly tilted easy axis extracted from the neutron scattering measurements.^[^ ^37^ ^]^ The total magnetic energy *U_M_* before and after the SF transition field *H_sf_* can be written as

(1) $$U_{M}=\left\{\begin{array}{c} -M_{0}H_{E},H_{⊥}<H_{sf}\;\\ M_{0}\left(H_{E}\cdot cos2\theta -2H_{⊥}\cdot cos\theta +H_{A}\cdot sin\theta^{2}\right),H_{⊥}\geq H_{sf} \end{array}\right.$$

where *M*_0_ is the sublattice magnetization, *H_E_* is the exchange field between the two spin sublattices.^[^ ^39^ ^]^ θ is the spin canting angle as illustrated in Figure 4a, *H_A_* is the magnetic anisotropy field, and *H*_⊥_ is the applied external magnetic field. In the ground state spin configuration, we have the spin canting angle θ to be $\cos\theta =\frac{H_{⊥}}{2H_{E}-H_{A}}\;$. The total energy of this spin‐NEMS coupled system contains the elastic energy, work done by the boundary, and the magnetic energy. We can identify the strain change, and therefore the lattice parameter modifications, that minimizes the total energy as a function of the magnetic field. The full derivation is shown in the Experimental Section. Below the *H_sf_*, *U_M_* is constant, yielding a constant strain ε_0_ and minimal magnetoelastic effect. Above the *H_sf_*, the strain ε(*H*) will gain a quadratic magnetic field dependence of $\frac{2H_{⊥}^{2}}{3Y{\left(2H_{E}-H_{A}\right)}^{2}}\frac{\partial H_{E}}{\partial \varepsilon }$. Comparing the estimated magnetoelastic effects with our measured results, the frequency plateau between −3 T and +3 T and the rapid overall decrease with increasing fields can be attributed to the SF transition. The decrease in frequency, and therefore a decrease in strain, above the *H_sf_* infers that $\frac{\partial H_{E}}{\partial \varepsilon }<0$. This is consistent with the expectation that spin exchange interactions decrease with a larger lattice separation.

The two sharp dips in the resonator's linear and nonlinear responses are thus beyond the prediction of the simple SF model. To identify their origins, we now focus on the spin configuration near the SF transition field. Previous neutron scattering experiments extracted a large exchange energy of μ_0_ *H_E_*  ≈  100 T and a small anisotropy energy of μ_0_ *H_E_*  ≈  0.06 T.^[^ ^34^, ^35^ ^]^ After reaching the *H_sf_*, the spin canting angle is calculated to have $\cos\theta =\frac{H_{⊥}}{2H_{E}-H_{A}}\;$. θ is therefore close to $\pm \frac{\pi }{2}$ and remains at this limit within our measured field range. As a result, above *H_sf_*, the collinear AFM spin rotates to effectively a collinear in‐plane spin alignment, with a large Neel component lying in the *ab* plane. However, minimal in‐plane magnetic anisotropy was identified or predicted in the previous neutron scattering measurements^[^ ^34^, ^35^ ^]^ and theoretical studies.^[^ ^42^ ^]^ The in‐plane Neel vector component therefore does not have a preferred orientation. This leads to degenerate in‐plane spin configuration, and we expect the formation of AFM domains above the SF transition field, as depicted in Figure 4a.

### 2.3 Theoretical Simulations

To verify this, the DW dynamics are evaluated by spin‐dynamics simulations at different fields. The Section S15 (Supporting Information) contains the description of simulation methods and detailed results. Figure 4d,e shows the snapshot of the simulated Neel vector orientations near the spin‐flop field (*H*_sf_), which reveals the rich dynamics of spin textures within nanoscale dimensions. Under an external field of 1.1 *H*_sf_ (Figure 4e), simulations show multiple DWs merging and rapid DW movements, in comparison with the slower DW dynamics at other fields like 0.8 *H*_sf_ (Figure 4d). The DW velocity can be estimated from the simulations, which peaks at *H_sf_* as shown in Figure S21 (Supporting Information). Around the SF transition, substantial amounts of spin waves are emitted throughout the media, which propagate with velocities of several km s^−1^ (Figure S21, Supporting Information). The rapid DW motions show a strong layer dependence in the thin‐film limit as a result of interlayer coupling. To estimate our measured devices’ DW dynamics, we examine the extrapolated trend in Figure S20 (Supporting Information), which yields > 600 ms^−1^ DW velocities for the measured device thicknesses. The corresponding frequency for DW lateral motion across the laser‐detected area is then hundreds of MHz or higher, indicating multiple DW moving across the resonator within one mechanical vibration cycle. It is also expected from the simulations (see Movies S1–S3, Supporting Information) that there are rapid domain emergence and annihilation within nanosecond timescales at the SF transition, which further increases the domain‐induced dynamic frequency.

We attribute the sharp *H*_1_ and *H*_2_ transitions to the rapid and collective magnetic DW motions. Magnetic domains introduce additional magnetoelastic effects, which can be estimated with an additional magnetic domain wall energy *U_DM_* in the total NEMS system energy. With the presence of one DW, $U_{DW}=Js^{2}\frac{\pi }{\sigma }$ where σ is the domain wall width, and the exchange energy *J*  = *H_E_* μ_*B*_.^[^ ^13^ ^]^ At a transition field with rapid magnetic domain fluctuations, we model the system response as having a large number *N*(*t*) of identical DWs, each carrying the energy of *U_DM_*. The magnetostriction of the fluctuating DW number *N*(*t*) can be evaluated by considering the time‐averaged 〈*N*〉 within one mechanical vibration cycle, which yields a strain change of $\varepsilon \left(DW\right)-\varepsilon =\frac{1}{3Y}\;\frac{\partial }{\partial \varepsilon }\left[\langle N\rangle \cdot J\right]$. Since $\frac{\partial H_{E}}{\partial \varepsilon }<0$, the presence of DW will cause a reduction in strain and a decrease in resonator frequency. The sharp frequency dips in Figure 2a thus originate from the strongly fluctuating DW dynamics. We note here that these types of AFM domains are conventionally difficult to detect due to the vanishing net magnetization. While it is possible to have magnetization at the DW, it is very challenging to perform nanoscale magnetometry with magnetic fields large enough to induce these transitions (>4 T) here.

The *H*_1_ transition that is close to the expected *H_sf_* can be understood as the emergence and collective quenching of DWs and spin textures. On the other hand, the *H*_2_ transition corresponds to the realignment of the in‐plane Neel vector and the magnetic domains. The magnetic hysteresis in both the linear and nonlinear resonator measurements provides strong evidence to support the above assignment and further elucidate the nature of the two transitions. In the linear response regime, as shown in Figure 2a, the gradual decrease in frequency with an increasing field across *H_sf_* corresponds to the emergence of multiple domains. In contrast, the sharp frequency dip and, therefore, a narrow transition width when sweeping the field down past *H_sf_*, infers a high 〈*N*〉 DW change and a collective quenching of magnetic domains. Remarkably, such hysteric behavior is only present if the applied field is swept past the *H*_2_ transition, which is most evident from the nonlinear resonator measurements shown in Figure 4b,c (see also Section S6, Supporting Information for the extraction from linear resonator measurements). The origin of domain realignment at *H*_2_ can be qualitatively understood when we consider the AFM Neel vector's tilting angle of 8° at the zero field.^[^ ^37^ ^]^ Under an out‐of‐plane magnetic field, the external field then has an effective projection perpendicular to the magnetic anisotropy direction, which eventually can assist in aligning the in‐plane Neel vector direction along the tilting direction at higher fields. It is worth mentioning that there is still no conclusive microscopic model to understand the tilting of the easy‐axis and the magnetic anisotropy in MnPS_3_. Several papers discussed the important roles of long‐range interactions, like dipole‐dipole interactions, as the possible origin of the anisotropy.^[^ ^43^, ^44^, ^45^ ^]^ It remains an open question to fully understand the magnetic anisotropy in MnPS3 theoretically and subsequently to understand the realignment of domains at 7 T.

### 2.4 Enhanced Mechanical Nonlinearity Coupled to Domain Dynamics

Finally, the abnormal Duffing nonlinearity at *H*_1_ further reveals the impacts of complex spin texture. Under a high RF driving force, most of the mechanical responses, as plotted in Figure 3b, show a stiffness hardening that is fitted with a cubic term *k*_3_ > 0. Surprisingly, the resonance backbone curve at *H*_1_ (Figure 4h) exhibits a composite nonlinear behavior from softening to hardening, and the fitting requires the fifth‐order term *k*_5_ *z* ^5^ in the spring restoring force. The fitted *k*_3_ has an abrupt sign flip, along with a significant enhancement in *k*_5_, as summarized in Figure 4f,g at *H*_1_. The sign flip and negative *k*_3_ (spring softening) at *H*_1_ is intriguing, because material‐related nonlinearity mechanisms, like geometric nonlinearity and nonlinear damping,^[^ ^41^ ^]^ lead to spring hardening instead of a softening. The abnormal nonlinearity here is correlated with the rapid and complex spatial spin textures. The fluid‐like DW motion from simulations near the SF transition potentially leads to nonlinear elastic mechanics due to the spatially varying and time‐fluctuating magnetostriction forces. The strong nonlinearity from the enhanced fifth‐order term *k*_5_ *z* ^5^ infers a more chaotic spin‐induced interaction and potentially fluid dynamics from the spin texture. At a sufficiently high drive level, the spring restoring force is dominated by the *k*_5_ *z* ^5^ high‐order term (Figure 4h), which suggests an increase in long‐range spin interactions. The back action from layer rippling and slipping^[^ ^46^ ^]^ under a high RF drive and, therefore, large displacement can further modify the interlayer spin configuration and nonlinear dynamics. An integrated microscopic model of the spin‐induced mechanical nonlinearity and corresponding nonlinear dynamics will need further theoretical investigation.

## 3 Conclusion

In summary, we have identified hidden magnetic transitions in 2D AFM MnPS_3_ due to the collective dynamics of magnetic domains through nanomechanical resonator measurements. The abnormal mechanical nonlinearity modulation near the spin‐flop field suggests nonlinear elastic and fluid‐like spin texture behavior. The method established herein can be implemented to explore other vdW or thin‐film systems hosting topologically non‐trivial spin textures such as skyrmions, vortices, etc. Our work paves the way towards mechanical manipulation of spin texture and harnessing the nonlinear coupling between the spin and lattice. The findings will inspire the next‐generation nanomechanical devices coupled to spin textures for both classical and quantum information technologies.^[^ ^6^, ^7^ ^]^

## 4 Experimental Section

### Sample Fabrication

Four drumhead resonators were fabricated using few‐layer MnPS_3_ membranes by employing all‐dry transfer techniques to avoid any contamination on the flakes. 23 nm Pt on 2 nm Ti is deposited and patterned onto a bare sapphire substrate to form local gates. 500 nm SiO_2_ and 20 nm Al_2_O_3_ are deposited as dielectric layer using plasma‐enhanced chemical vapor deposition and atomic layer deposition techniques. Microtrenches with 520 nm depth and varying diameters are formed using reactive ion etching. 30 nm Au on 5 nm Ti are deposited for source and drain electrodes and 200 nm Au on 5 nm Ti are deposited for contact pads. Finally, exfoliated MnPS_3_ flakes are transferred to make the NEMS resonators (for additional information, see Section S1, Supporting Information). Atomic force microscopy was used to estimate the thickness of the transferred flakes (for details, see Section S1, Supporting Information).

### NEMS Measurement Setup

To precisely measure the resonance characteristics of the MnPS_3_ membranes, a custom‐built ultrasensitive optical interferometry system was engineered and optimized with a low‐power 633 nm laser for readout of device motion (Figure 1c). Such interferometry system is robust and can probe mechanical resonances both in linear and nonlinear regimes. A DC voltage from a source meter (Keithley 2400) was combined with an RF voltage from a network analyzer (HP3577A) using a bias‐tee, and is applied to the local gate to drive the resonator. The interferometric signal contains information of the fm‐ to pm‐scale vibrations of the membranes. A photodetector converts the optical signal to electronic signal that is read out by the network analyzer. More details are provided in Section S3 (Supporting Information). Linear resonance measurement is performed at low RF voltage (≈10 mV) and the nonlinear resonance characterization was performed at 400 mV. Initial device characterization is done at room temperature and in moderate vacuum (≈20 mTorr). Magnetic field dependency is carried out at cryogenic temperature (≈4 K) with the Attodry 1000 system (at the University of Florida) and also partially done at the National High Magnetic Field Laboratory in Tallahassee, Florida.

### Resonance Frequency Tuning and Estimation of Young's Modulus

Resonance frequency tuning of the fundamental mode of a drumhead resonator with initial strain ε_0_, Poisson's ratio ν, depth of the air gap *h*_0_, and effective mass *m*_eff_ =  0.2695π*r* ^2^ *h*ρ can be described by:^[^ ^47^ ^]^

(2) $$f_{0}\left(V_{g}\right)=\frac{1}{2\pi }\sqrt{\frac{\frac{\pi \varepsilon_{0}^{2}r^{2}}{8\left(1-\nu^{2}\right)E_{Y}h\varepsilon_{0}^{2}h_{0}^{4}}{\left(V_{g}-V_{0}\right)}^{4}+4.9E_{Y}h\varepsilon_{0}-\frac{\varepsilon_{0}\pi r^{2}}{3h_{0}^{3}}{\left(V_{g}-V_{0}\right)}^{2}}{m_{\mathrm{eff}}}+\frac{\beta_{0}^{4}D}{\rho hr^{4}}}$$

Here, for the first mode, $\beta_{0}^{2}$= 10.215. Because of charge trapping in the suspended flake, the charge neutrality point shifts during the gate voltage sweeps, and *V*_0_ accounts for this effect. Young's modulus (*E*_Y_) and the volume mass density (ρ) of the membrane can be found by fitting the experimentally measured resonance with varying gate voltage. The extracted values for resonator 1 are *E*_Y_ = 770 GPa, ε_0_ = 0.0008, and ρ = 2.5ρ_0_. The volume mass density is 2.5 times higher than that of the expected value (ρ_0_ = 2920 kg m^−3^) likely due to the presence of surface adsorbates on the membrane at cryogenic temperatures. The extracted values for resonator 2 are *E*_Y_ = 1659 GPa, ε_0_ = 0.0007, and ρ = 5.5ρ_0_ and resonator 4 are *E*_Y_ = 485 GPa, ε_0_ = 0.0018, and ρ = 3.8ρ_0_. More details about fitting can be found in Section S8 (Supporting Information).

### Magnetostriction in Spin‐Flop Transition

The total energy of the magnetic membrane system *U* contains contributions from the magnetic terms. *U_M_*, elastic energy *U_el_* and the work done by the boundary *U_b_*, *U*  = *U_M_*  + *U_el_* + *U_b_*. $U_{el}=\frac{3}{2}\;E_{Y}\varepsilon^{2}$, where *E_Y_* is the effective Youn's modulus and ε is the strain, and *U_b_* =   − 2*U_el_*.^[^ ^32^ ^]^ were used. To estimate the magnetostriction of an SF transition, the magnetic energy of AFM to canting spin states (Figure 4a inset) was considered with a canting angle of θ. In the AFM phase, *U_M_* =   − *H_E_M*_0_, where *H_E_* is exchange field and *M*_0_ is the magnetization of a sublattice spin. In the spin‐flop (canted) phase with a magnetic field of *H* parallel to the easy axis, *U_M_* = *H_E_* *M*_0_ *cos*2θ − 2*HM*_0_ *cos*θ + *H_A_M*_0_sin ^2^θ. Since $cos\theta =\frac{H}{2H_{E}-H_{A}}\;,$ the magnetic energy can be further simplified to $U_{M}=\left(-H_{E}+H_{A}-\frac{H^{2}}{2H_{E}-H_{A}}\right)\;M_{0}$. The SF field can be solved: $H_{sf1}=\sqrt{H_{A}\left(2H_{E}-H_{A}\right)}\;$ and $H_{sf2}=\sqrt{H_{A}\left(2H_{E}+H_{A}\right)}\;$. Since *H_A_* ≪ *H_E_*, their differences can be ignored, and the magnetic energy change is continuous from the AFM to the SF state.

The strain ε as a function of the magnetic field can be found out by minimizing the total energy.$\;\frac{\partial U}{\partial \varepsilon }=0$, which yields $-3E_{Y}\varepsilon +\frac{\partial U_{M}}{\partial \varepsilon }=0$. Since *U_M_* does not depend on the magnetic field in the AFM state, the strain is a constant. In the SF state, the strain change compared to the AFM state is then $\frac{3E_{Y}}{M_{0}}\;\left(\varepsilon_{AFM}-\varepsilon_{SF}\right)=-\frac{\partial H_{A}}{\partial \varepsilon }\left(1-\frac{H^{2}}{{\left(2H_{E}-H_{A}\right)}^{2}\;}\right)-\frac{\partial H_{E}}{\partial \varepsilon }\frac{2H^{2}}{{\left(2H_{E}-H_{A}\right)}^{2}}\approx =-\frac{\partial H_{A}}{\partial \varepsilon }-\frac{\partial H_{E}}{\partial \varepsilon }\frac{2H^{2}}{{\left(2H_{E}-H_{A}\right)}^{2}}$

### Estimation of Responsivity and Fitting of Nonlinear Resonance Signals

The critical amplitude (*a*_c_) for onset of nonlinearity of a drumhead resonator is given by $a_{c}=\sqrt{\frac{8\sqrt{3}}{9k_{3}^{2}Q}}\;$, where the Duffing coefficient can be estimated by $k_{3}^{2}=\frac{13+21\nu -4\nu^{2}}{30\left(1+\nu \right)r^{2}\varepsilon_{0}}\;$. *a*_c_ = 0.91 nm is estimated at 4 K and 0 T. From the nonlinearity measurement, it also estimates the signal caused by the critical amplitude *v*_c_ = 58 µV in the voltage domain. From these two, the displacement‐to‐voltage responsivity (transduction gain) of the fundamental mode is obtained to be 64 nV pm^−1^. The backbone curve of the nonlinear response can be expressed as^[^ ^48^ ^]^

(3) $$\Omega =\omega_{0}+\frac{3k_{3}z_{0}^{2}}{8\omega_{0}m_{\mathrm{eff}}}+\frac{5k_{5}{z_{0}}^{4}}{16\omega_{0}m_{\mathrm{eff}}}$$

where *k*_3_ and *k*_5_ denote the Duffing and quintic nonlinear coefficients, *Ω*, *m*_eff_, and *z*_0_ represent the instantaneous resonance frequency, effective mass, and amplitude, respectively. Using this equation, *k*_3_ and *k*_5_ were estimated as fitting parameters from the calibrated displacement measurement. Estimates *k*_3_ and *k*_5_ were also obtained directly in the voltage domain without the displacement calibration with units N/V^3^ and N/V,^5^ respectively. The trend of *k*_3_ and *k*_5_ matches qualitatively in both voltage and displacement domains.

### Atomistic Spin Dynamics

Spin dynamics simulations methods^[^ ^49^, ^50^, ^51^, ^52^, ^53^ ^]^ were used to calculate the magnetic properties of MnPS_3_. The system was described using the spin Hamiltonian:

(4) $$H=-\frac{1}{2}\sum_{i\neq j}J_{ij}S_{i}\cdot S_{j}-\frac{1}{2}B\sum_{\mathrm{NN};i\neq j}{\left(S_{i}\cdot S_{j}\right)}^{2}-K\sum_{i}{\left(S_{i}^{z}\right)}^{2}$$

where *S*_*i*,*j*_ are unit vectors representing the local spin directions on sites *i,j*. The first term represents bilinear exchange with the exchange constant *J_ij_* between spins *i* and *j*. The bilinear exchange was considered up to the third nearest neighbor for in‐plane and out‐of‐plane neighbors. The second term describes the biquadratic exchange contribution between sites *i* and its in‐plane nearest neighbors and *B* is the biquadratic exchange constant. Both the bilinear and biquadratic interactions are isotropic. The last term is the easy‐axis anisotropy contribution with the magnetocrystalline constant *K*. Table S2 (Supporting Information) gives the exchange parameters used for the calculations. Additional details are included in Section S15 (Supporting Information).

## Conflict of Interest

The authors declare no conflict of interest.

## Author Contributions

X.‐X.Z. and P.F. conceived the experiments. S.E.Y. and Y.W. fabricated the devices and performed data analysis. S.E.Y., Y.W., and J.K‐P. performed the experiments. L.S., S.M., C.T., and D.S. assisted in the measurements done in the National High Magnetic Field Lab. E.J.G.S. designed the theoretical approach. S.R. performed spin dynamics simulations under E.J.G.S. supervision. X.‐X.Z. and S.E.Y. wrote the manuscript with critical inputs from P.F., E.J.G.S., and S.R. All authors discussed the results and commented on the manuscript.

## Supporting information



Supporting Information

Supplemental Movie 1

Supplemental Movie 2

Supplemental Movie 3

## Data Availability

The data that support the findings of this study are available from the corresponding author upon reasonable request.
