# Supplementary material for: Mechanical Resonant Sensing of Spin Texture Dynamics in a 2D Antiferromagnet
Source: Adv Mater. 2025 Apr 30;37(29):2420168. doi: 10.1002/adma.202420168 (PMC12288789; doi:10.1002/adma.202420168)
Supplement: Supplementary file 1 — Supporting Information [file ADMA-37-2420168-s002.pdf]

ADVANCED MATERIALS

Supporting Information

for *Adv. Mater.*, DOI 10.1002/adma.202420168

Mechanical Resonant Sensing of Spin Texture Dynamics in a 2D Antiferromagnet

S M Enamul Hoque Yousuf, Yunong Wang, Shreyas Ramachandran, John Koptur-Palenchar, Chiara Tarantini, Li Xiang, Stephen McGill, Dmitry Smirnov, Elton J. G. Santos, Philip X.-L. Feng* and Xiao-Xiao Zhang**

Supporting Information

Mechanical resonant sensing of spin texture dynamics in a two-dimensional antiferromagnet

S M Enamul Hoque Yousuf[1], Yunong Wang[1], Shreyas Ramachandran[2], John Koptur-Palenchar[3], Chiara Tarantini[4], Li Xiang[4], Stephen McGill[4], Dmitry Smirnov[4], Elton J. G. Santos[2,5,6], Philip X.-L. Feng[1,3]*, Xiao-Xiao Zhang[3]**

[1]Department of Electrical & Computer Engineering, University of Florida, Gainesville, Florida 32611, USA

[2]Institute for Condensed Matter Physics and Complex Systems, School of Physics and Astronomy, The University of Edinburgh, Edinburgh EH9 3FD, UK

[3]Department of Physics, University of Florida, Gainesville, Florida, 32611, USA

[4]National High Magnetic Field Laboratory, Tallahassee, 32312, Florida, USA

[5]Donostia International Physics Centre (DIPC), Donostia-San Sebastian, 20018, Spain.

[6]Higgs Centre for Theoretical Physics, The University of Edinburgh, EH9 3FD, UK

[*]Emails: xxzhang@ufl.edu, philip.feng@ufl.edu, esantos@ed.ac.uk

Table of Contents

S1 Fabrication and Thickness Measurements of $MnPS_3$ Drumhead Resonators 2

S2 Magnetization Measurements of Bulk $MnPS_3$ 3

S3 Laser Interferometry System 4

S4 Theoretical Analysis of the $MnPS_3$ Drumhead Resonator and Frequency Scaling 5

S5 Additional Data on Magnetic Field Dependency of Linear Resonance 6

S6 Hysteresis in Linear Resonance 8

S7 Effect of Magnetic Field on Quality Factor 9

S8 Resonance Frequency Tuning in $MnPS_3$ Resonators 10

S9 Nonlinear Characterization of $MnPS_3$ NEMS Resonators 11

S10 Additional Data on Nonlinear Characterization at Varying Magnetic Fields 13

S11 Frequency Stability 14

S12 Measured Resonance Response at 100 K 15

S13 Estimation of Strain Change 15

S14 Summary of Measured Results 16

S15 Simulation Methods and Results 16

S1 Fabrication and Thickness Measurements of $MnPS_3$ Drumhead Resonators

To minimize unwanted RF signal crosstalk, we fabricate electrostatically tunable $MnPS_3$ NEMS resonators by utilizing a sapphire wafer, ensuring pristine device performance at both room temperature and 4 K (Fig. S1). The fabrication process initiates by patterning 23 nm Pt on 2 nm Ti local gates for precise electrostatic control. Subsequently, we deposit a 500 nm SiO_2 and 20 nm Al_2O_3 dielectric layer via plasma enhanced chemical vapor deposition (PECVD) and atomic layer deposition (ALD), respectively, to ensure robust electrical isolation. We then form microcavities of varying diameters using reactive ion etching (RIE). Next, we deposit 30 nm Au on 5 nm Ti for source and drain electrodes, and 200 nm Au on 5 nm Ti for contact pads. Finally, we transfer few-layer mechanically exfoliated $MnPS_3$ flakes onto the prefabricated substrates using an all-dry transfer method[1]. For room temperature measurement, we wire bond the devices with a ceramic package using gold wire. For low temperature measurement, we utilize indium to connect the gold wires between the contact pads and the designed low temperature package. The additional electrodes in devices 1 and 2 (marked in the Fig. S1g) can be used to modulate the contact between the $MnPS_3$ and gold electrodes. However, for the current set of experiments, we did not use these electrodes.

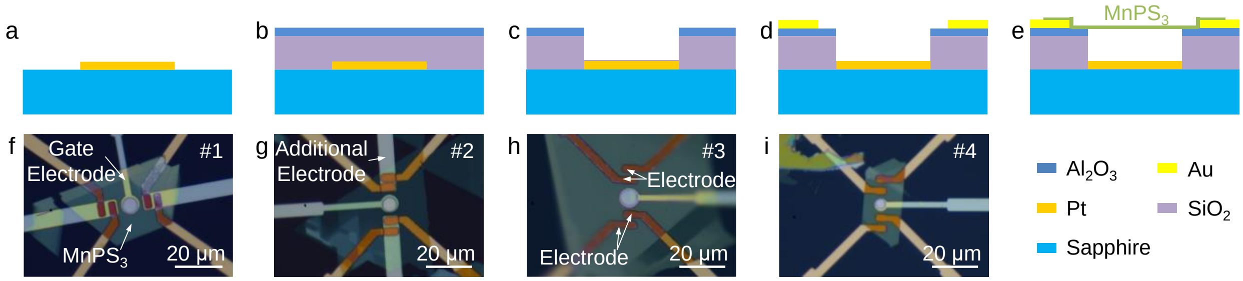

Figure S1| Fabrication process of $MnPS_3$ NEMS resonators. **a,** Patterning local gate. **b,** Depositing SiO_2 and Al_2O_3. **c,** Forming microcavities. **d,** Depositing metal electrodes and contact pads. **e,** Transferring few-layer $MnPS_3$ flakes onto the substrate. **f- i,** Optical image of the measured $MnPS_3$ NEMS resonators. The number on the top right corner with # indicates the device identification number. Scale bar: 20 μm.

Utilizing a Digital Instruments Dimension 3100 atomic force microscopy in tapping mode, we acquire atomic force microscopy images to ascertain the thickness of the few-layer $MnPS_3$ flakes. High-resolution scans, following initial coarse scans that center the device within the scan field, enable us to capture detailed images of $MnPS_3$ flake portions that appear similar in color to the suspended sections. To avoid potential damage from the atomic force microscopy tip, we carefully avoid contacting the suspended areas. The thickness is determined by averaging the height variations across multiple traces along the flake's edge. Figure S2 shows the measured thickness of the 4 devices used in this work.

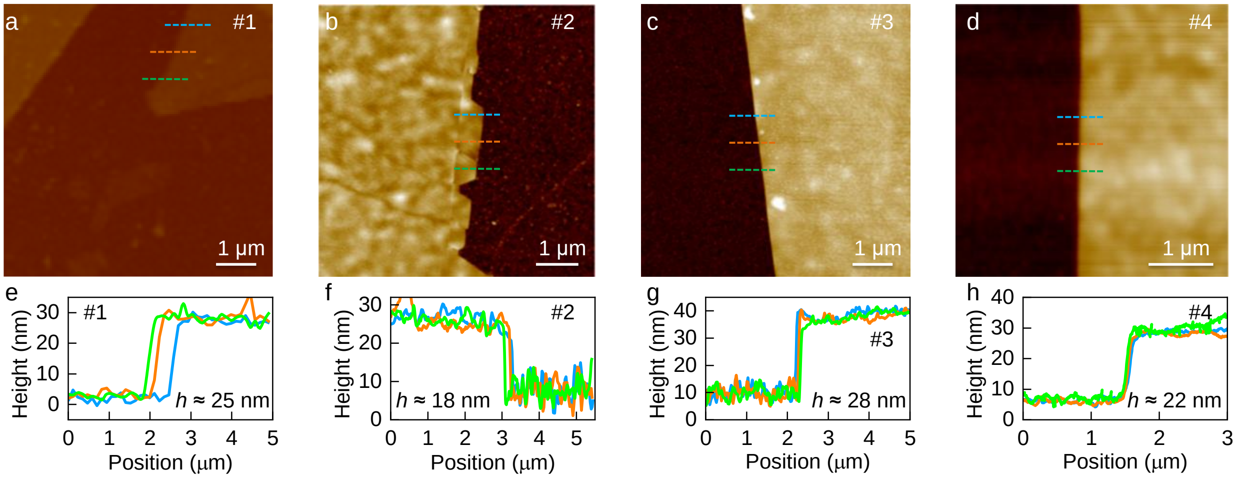

Figure S2| Atomic force microscopy measurement to determine the thickness of the $MnPS_3$ flakes. **a-d,** Atomic force microscopy scan of the flakes used in devices 1-4. **e-h,** Height traces from the atomic force microscopy image along the lines shown in (**a**)-(**d**). The color of the traces in (**e**)-(**h**) corresponds to the cut lines in (**a**)-(**d**).

S2 Magnetization Measurements of Bulk $MnPS_3$

Magnetic characterization was performed in a Quantum Design (QD) 16 T Physical Property Measurement System (PPMS) equipped with a Vibrating Sample Magnetometer (VSM). Several crystals were stacked together to increase the signal. The crystals were installed in a QD brass trough sample-holder and secured with vacuum grease between two quartz braces, which maintain the crystals in perpendicular configuration. The magnetic measurement was performed at 4.0 K by increasing the magnetic field from 0 to 10 T, decreasing it down to -10 T, and finally increasing the magnetic field to 0 T.

The magnetic moments of the measured bulk $MnPS_3$ crystals is plotted in Fig. S3a. Fig. S3b plots the corresponding first-order derivative (proportional to magnetic susceptibility). The spin-flop field is assigned to be the point with the most rapid increase rate of magnetization as a function of fields (maximum in the first-order derivative of M), which is ~4.7T in our crystal, also consistent with previous results. There is no obvious magnetization transition at 7T from both our measurements and the previous report. To further compare with our results in the mechanical responses as a function of field, the magnetization plot is sorted and colored differently for the direction of scanning from the low-to-high field (0→10T and 0 →-10T) in black, and the high-to-low field (10 → 0T and -10 → 0T). The comparison shows that there is obvious hysteretic behavior in magnetization, which is consistent with prior reports in magnetization measurements and neutron scattering. It thus again highlights the different origins of sharp hysteretic transitions near H_{sf} observed in mechanical responses in the main text.

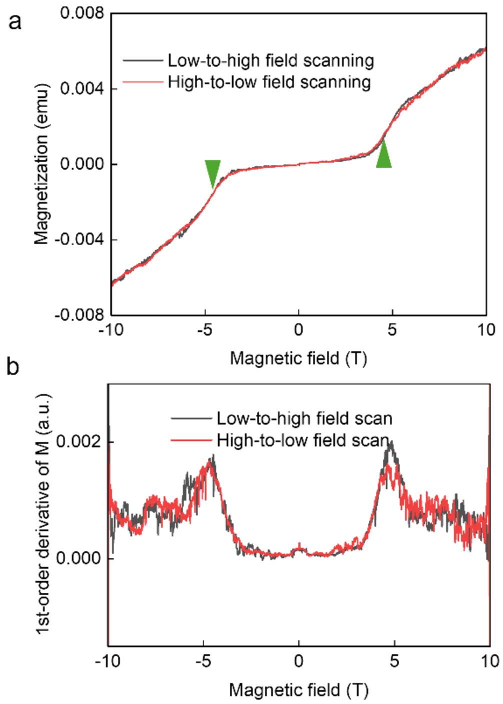

Figure S3| (a) The magnetization as a function of out-of-plane magnetic field at 4K measured in the bulk crystals used for device fabrication in our report. The different colors represent different field scanning as described above in the response. The green triangle indicates the field with the fastest change rate of magnetization, corresponding to the maximum magnetization first-order derivative plotted in (b).

S3 Laser Interferometry System

We characterize the resonators using a custom-built optical interferometry system, incorporating a 633 nm He-Ne laser. We use low laser power (<12 μW) to prevent laser heating at 4 K. We use a 100× objective lens to achieve a laser spot approximately below 1 μm in diameter, positioned at the center of the suspended device to maximize the probe signal. For room temperature measurement, the device is mounted onto a ceramic package and placed inside a vacuum chamber equipped with SMA connectors and an optical window. For low temperature measurement, the device is mounted on a printed circuit board (PCB) and loaded inside a cryostat. A DC voltage from a source meter (Keithley 2400) is combined with an RF signal from a network analyzer (HP3577A) using a bias-tee (from Mini-Circuits) and is applied to the local gate of the resonator. The interference among the reflected lights from vacuum-$MnPS_3$, $MnPS_3$-vacuum, and vacuum-Pt generates the interferometric signal which contains the vibration information of the membrane. A photodetector converts the optical signal to electrical signal and read out by the same network analyzer. The cryostat contains DC magnet, and the magnetic field can be swept from 0 T to higher magnetic fields. To measure the frequency stability of the resonator and calculate Allan deviation, we use the phase locked loop module of Zurich Instruments (UHFLI 600MHz Lock-in Amplifier).

S4 Theoretical Analysis of the $MnPS_3$ Drumhead Resonator and Frequency Scaling

The resonance frequency (f_0) of a uniform drumhead resonator with radius r (m), volume mass density of the 2D material ρ (kg/m^3), thickness h (m), flexural rigidity D (N·m), and tension γ (N/m) is given by [2]

$$f_0 = \frac{1}{2\pi}(k_2^{mn}r)\sqrt{\left(\frac{D}{\rho h r^4}\right)\left[(k_2^{mn}r)^2 + \frac{\gamma r^2}{D}\right]} \quad (S1)$$

The flexural rigidity D is given by $D=E_Y h^3/[12(1-\nu^2)]$, where E_Y is the Young's modulus and ν is Poisson's ratio. In Eq. S1, $k_2^{mn}r$ is a mode dependent parameter and determined numerically. The value of $k_2^{mn}r$ can be approximated by the following double exponential function [2]

$$(k_2^{mn}r)^2 = f\left(\frac{\gamma r^2}{D}\right) = \alpha_{mn} + (\beta_{mn} - \alpha_{mn})\exp\left[-c_1 \exp\left\{c_2 \ln\left(\frac{\gamma r^2}{D}\right)\right\}\right] \quad (S2)$$

where c_1 and c_2 are used as fitting parameters to minimize the errors between theory and the approximation given by Eq. S2. The parameters α_{mn} and β_{mn} are calculated numerically and for the lowest mode (fundamental mode) $\alpha_0 = 5.7832$ and $\beta_0 = 10.215$ with c_{10}=0.1148 and c_{20}=0.4868. In the ideal membrane limit, tension dominates and $\gamma d^2/D = \infty$. From Eq. S2, $(k_2^{mn}r)^2 = f(\infty) = \alpha_0 = 5.7832$, and Eq. S1 reduces to

$$f_0 = \frac{2.405}{2\pi r}\sqrt{\frac{\gamma}{\rho h}} \quad (S3a)$$

Therefore, in the membrane limit, the resonance frequency is set by the tension on the membrane. Modulating the tension in the membrane thus provides a control knob to tune f_0 in the NEMS resonators operating in the membrane regime. The resonance frequency f_0 scales with the radius and thickness according to $r^{-1}h^{-0.5}$. In the ideal plate limit, flexural rigidity dominates and $\gamma d^2/D$ = 0. From Eq. S2, $(k_2^{mn}r)^2 = f(0) = \beta_0 = 10.215$, and Eq. S1 reduces to

$$f_0 = \frac{10.215}{2\pi r^2}\sqrt{\frac{D}{\rho h}} = \frac{10.215}{2\pi}\sqrt{\frac{E_Y}{12\rho(1-\nu^2)}}\frac{h}{r^2} \quad (S3b)$$

Therefore, in plate regime, f_0 scales with h/r^2. Resonance frequency depends on both tension and flexural rigidity in the transition regime between the two extreme cases of ideal membrane and plate. We calculate and plot f_0 for $MnPS_3$ NEMS resonators at room temperature as a function of number of layers for different built-in tension (Fig. S4). f_0 in the fabricated devices, however, also depends on other factors, such as clamped boundaries, built-in tension from the polydimethylpolysiloxane (PDMS) transfer, and temperature during the transfer process.

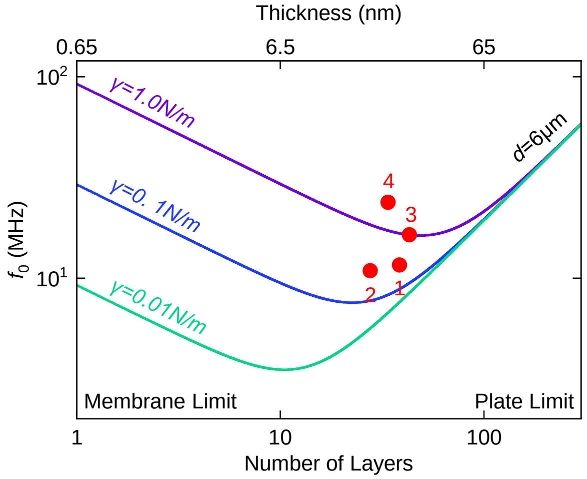

Figure S4| Frequency scaling of $MnPS_3$ NEMS resonators. Data points are calculated using Eq. S1 with E_Y=85 GPa, ν=0.327, and ρ=2920 kg/m^3.[3,4] Red filled circles represent the measured resonance in devices 1-4 at room temperature (d_1=d_2=6μm, d_3=7μm, d_4=4μm).

S5 Additional Data on Magnetic Field Dependency of Linear Resonance

To investigate the effect of magnetic field on the resonance frequency, we measure and characterize $MnPS_3$ resonators with different diameter and thickness. Fig. S5a shows the magnetic field dependence of the resonance frequency in device 1 without accounting for the time drift in f_0. A complete description of the trend of f_0 in device 1 can be found in the Main Text. Fig. S5b shows the trend of f_0 in device 2 for different magnetic fields. For sweeping the magnetic field, we have followed the same protocol in device 1 (0 T to 9 T to -9 T to 0 T). The transition magnetic fields are at $\mu_0 H_1$=±4.1 T and $\mu_0 H_2$=±6.7 T, which is very clear from the dip observed at those magnetic fields. The dip is more apparent from high magnetic field to low magnetic field sweep, which concurs with the observation in device 1. Additional characterization in a thicker device with h=28 nm demonstrates $\mu_0 H_1$=5.5 T and $\mu_0 H_2$=7.2 T (Fig. S5c). Different parts of the magnetic field sweeps in device 3 are performed on different days and hence the overall trend of the four segments of the magnetic field sweep looks different compared to device 1 and 2. However, the two dips at H_1 and H_2 are clearly observed for this thicker sample as well. Device 4 with h=22 nm demonstrates the first dip at $\mu_0 H_1$=4.1 T (Fig. S5d). The second dip is not visible in this device due to a large step during magnetic field sweep near the second transition. Table 1 summarizes the measured results in different devices.

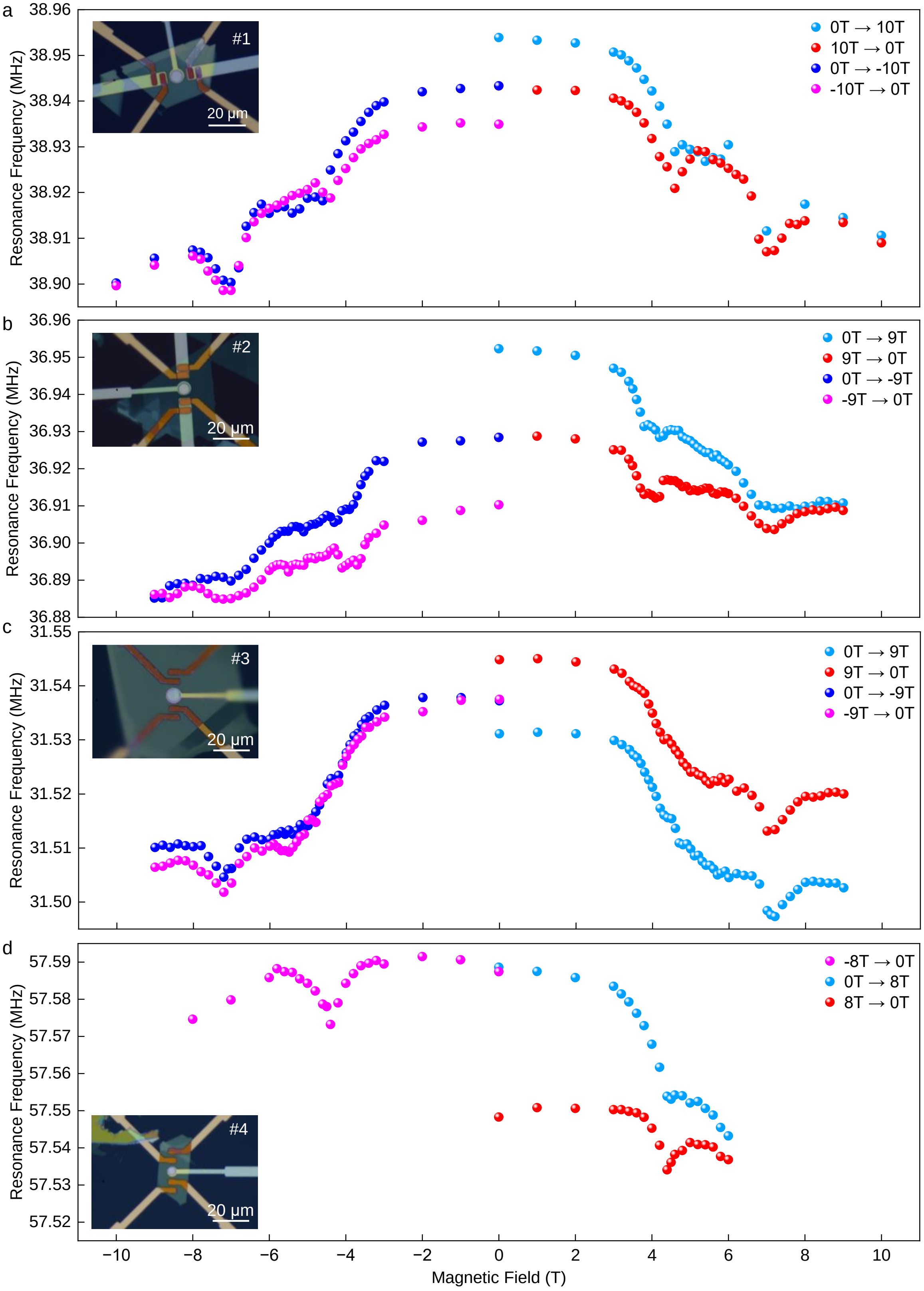

Figure S5| Measured resonance frequency as a function of out-of-plane magnetic field in device **a,** 1, **b,** 2, **c,** 3, and **d,** 4, respectively. Inset shows the optical image of the devices.

S6 Hysteresis in Linear Resonance

To demonstrate the effect of the second transition on the first transition, we perform a controlled experiment where we switch the sweep direction of *H* at certain values. As stated earlier, the dip in the resonance frequency is more prominent when we sweep *H* from high to low value. As shown in Fig. S6a, the change in f_0 around the dip is 2.1 kHz when we sweep from 0 T to 9 T. However, this change in f_0 increases to 4.3 kHz when we sweep back from 9 T to 0 T. The complete data for the 0 T to 9T is shown in Fig. S5a. We hypothesize that the change in the spin orientation due to the second transition affects the first transition when we sweep from high to low magnetic field. To confirm our hypothesis, we increase the magnetic field from 0 T to 5.3 T and change the sweep direction back towards 0 T (Fig. S6b). The change in f_0 around the dip during sweeping from 5.3 T to 0 T is ~2.6 kHz. Therefore, we conclude that the first transition depends on the second transition. If we stop sweeping *H* before we reach the second transition, dip is not as sharp as we observe compared to when we increase *H* to take place the second transition.

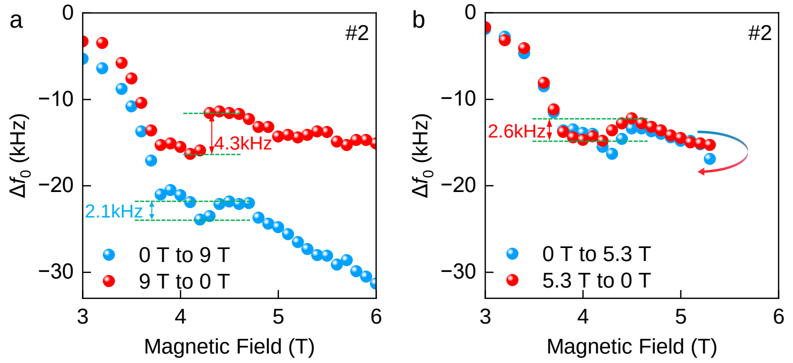

Figure S6| Effect of the second transition on the first transition. **a,** Magnetic field is swept from 0 T to 9 T and then swept back to 0 T. **b,** Magnetic field sweep direction changed towards 0 T before the second transition took place.

S7 Effect of Magnetic Field on Quality Factor

The quality factor of the drumhead resonator varies with the applied external magnetic field. Figure S7a shows extracted Q in device 2 measured at 4 K under varying H. The resonator's quality factor shows clear dips at both transition fields. At H=0 T, the resonator exhibits $Q \approx 5000$ with a moderate signal amplitude at v_{RF}=50 mV. At this drive level, the frequency domain response displays a Lorentzian shape, indicating that the dissipation can be modeled by a finite Q. As $\boldsymbol{B}$ is swept from 0 T to 9 T, Q decreases near the first transition to ~4000. Once the first transition is completed, the Q of the resonator increases, returning to ~5000. However, near the second transition, Q decreases again to ~4000 and goes back to ~5000 once the second transition is completed. This indicates that the resonator experiences strong dissipation near both transition fields. The trend of the intensity of the signal is shown in Fig. S7b. Near/at the transition fields, the intensity of the response diminishes with broadened linewidth. However, the trend in intensity is not as clear as the trend in f_0 and Q.

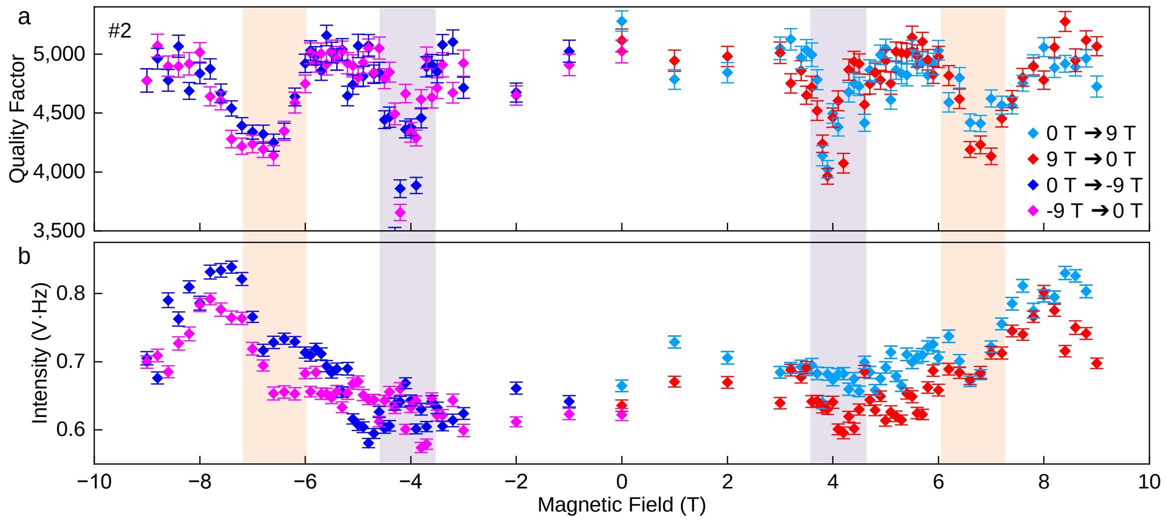

Figure S7| Effect of spin orientation on the dissipation of the resonator. **a,** Change in Q and **b,** intensity of the measured frequency domain response from device 2. Clear dips in both Q and amplitude are observed from the measurement.

S8 Resonance Frequency Tuning in $MnPS_3$ Resonators

With the application of V_g at the bottom gate, the membrane is stretched towards the gate due to electrostatic force and thus the tension in the membrane increases with increased V_g. However, increased V_g also introduces capacitive softening which reduces the resonance frequency. Thus, the frequency tuning of the drumhead resonator depends on the interplay between the V_g-induced elastic stiffening and capacitive softening effect. The electrostatic tuning of a drumhead resonator can be expressed as [5,6]

$$f_0(V_g) = \sqrt{f^2_{\mathrm{membrane+capacitance}} + f^2_{\mathrm{plate}}}$$

$$= \frac{1}{2\pi}\sqrt{\frac{\frac{\pi\epsilon_0^2 r^2}{8(1-\nu^2)E_Y h\varepsilon_0^2 h_0^4}\left(V_g - V_0\right)^4 + 4.9E_Y h\varepsilon_0 - \frac{\epsilon_0 \pi r^2}{3h_0^3}\left(V_g - V_0\right)^2}{m_{\mathrm{eff}}} + \frac{\beta_0^2 D}{\rho h r^4}} \qquad \text{(S4)}$$

where ε_0, r, h_0, ϵ_0, and $m_{\mathrm{eff}} = 0.2695\pi r^2 h\rho$ represent initial radial strain due to stretching of the membrane, radius of the drumhead, depth of the air gap (trench depth), permittivity of the free space, and effective modal mass for the first mode, respectively. Here, for plate regime, β_0=10.215. V_0 accounts for the shift of the charge neutrality point possibly due to charge trapping during the gate voltage sweeping. Depending on the initial device parameters, such as built-in tension and vacuum (or air) gap between the suspended membrane and the local gate of the resonator, the combined effect can lead to *W*, *U*, or reversed *U* shaped frequency tuning curves. Figure S8 shows the tuning curves of f_0 measured at 4 K by varying V_g between ±20 V. By fitting the data points to Eq. S4, we extract the initial strain of the devices at 0 T. Note that increasing the gate voltage sweep range provides the *W* shaped tuning curve where we can see the inversion point when the gate induced tensioning of the membrane starts to dominate.

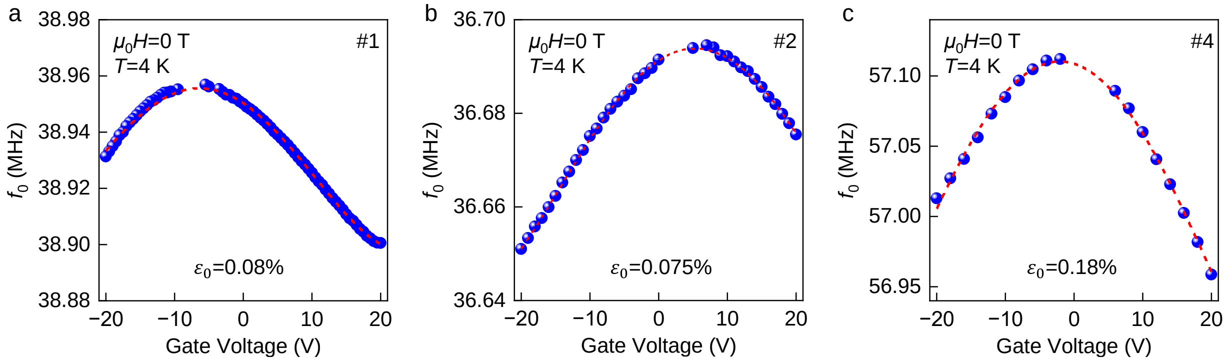

Figure S8| Resonance frequency tuning via gate voltage. **a-c,** Resonance frequencies at varying gate voltages between -20 V to 20 V measured from device 1, 2, and 4, respectively. The red dashed curves are fitted to Eq. S4.

S9 Nonlinear Characterization of $MnPS_3$ NEMS Resonators

To characterize the resonance motion of the device driven into nonlinearity, we consider a single degree of freedom model of a driven damped harmonic resonator with its eigen frequency $\omega_0 = 2\pi f_0$, linear damping term ($\omega_0 \dot{x}/Q$), linear restoring force ($k_{eff}\, x = m_{eff}\, \omega_0^2 x$), Duffing ($k_3\, x^3$), and quintic nonlinearity term ($k_5\, x^5$). The equation governing the resonator's motion can be expressed as follows

$$m_{\mathrm{eff}}\ddot{x} + \frac{m_{\mathrm{eff}}\omega_0\dot{x}}{Q} + k_{\mathrm{eff}}x + k_3x^3 + k_5x^5 = F_{\mathrm{ext}} \qquad (S5)$$

Here, m_{eff}, k_3, and k_5 represent the effective mass, cubic nonlinear coefficient, and quintic nonlinear coefficients, respectively. $F_{ext} = F\cos(\omega t)$ describes the external driving force with angular frequency $\omega = 2\pi f$. The nonlinear stiffness components associated with k_3 and k_5 account for the amplitude-dependent frequency shift at higher driving voltage and the corresponding bending of the frequency response curve near the resonance. By employing method of averaging to capture the slow variations of the amplitude and phase response of the resonator, the relation between the instantaneous frequency (Ω) and amplitude (z_0) can be expressed as [7]

$$\Omega = \omega_0 + \frac{3k_3z_0^2}{8\omega_0 m_{\mathrm{eff}}} + \frac{5k_5z_0^4}{16\omega_0 m_{\mathrm{eff}}} \qquad (S6)$$

Here, Eq. S6 delineates the backbone curve of the nonlinear response of the resonator and can be used to estimate the nonlinear stiffness parameters k_3 and k_5 as fitting parameters in experimentally measured results. Depending on the direction of the drive frequency sweep in a Duffing resonator, the resonance response is different and causes a frequency hysteresis in the resonator responses. We use the frequency hysteresis (Δf_{hys}) to confirm the first and second transition fields in the $MnPS_3$ resonators. We note that the damping terms were not considered in the extraction of nonlinear coefficients.

We measure the nonlinear response of device 2 both at room temperature and at 4 K. At room temperature, the resonator shows stiffening nonlinearity with higher v_{RF} (V_{DC}=15 V). Operating the resonator at low temperature increases f_0 and Q in all the tested devices. At higher drive voltage (v_{RF}), device 2 shows stiffening nonlinearity. We characterize the nonlinear response with both forward and backward sweeps and calculate the frequency hysteresis (Δf_{hsy}). Figure S8a-8d show the forward sweep at varying magnetic fields. A sharp drop in amplitude can be seen at the first transition field. At H_1, the forward and backward frequency sweeps also coincide with each other and the frequency hysteresis disappears. We use Eq. S6 to estimate the cubic and quintic nonlinear coefficients at varying magnetic fields. Figure S8e-8h show the backbone curves at 3.8, 4.2, 5.2, and 6.7 T, respectively. We observe that k_3 goes from stiffening to softening at the first transition field. k_3 also becomes smaller compared to its neighboring fields for the second transition field H_2. The quintic nonlinear coefficients, on the other hand, stays insignificant in all cases except near the critical fields. Since the quintic term is related to fifth order nonlinearity, the contribution to the overall force term is negligible at 0 T. However, near the first critical field, the quintic term increases significantly, leading to a flame curve (Fig. S9f). The overall trends of k_3 and k_5 are shown in Fig. S10 when we sweep the magnetic field from 9 T to 0 T.

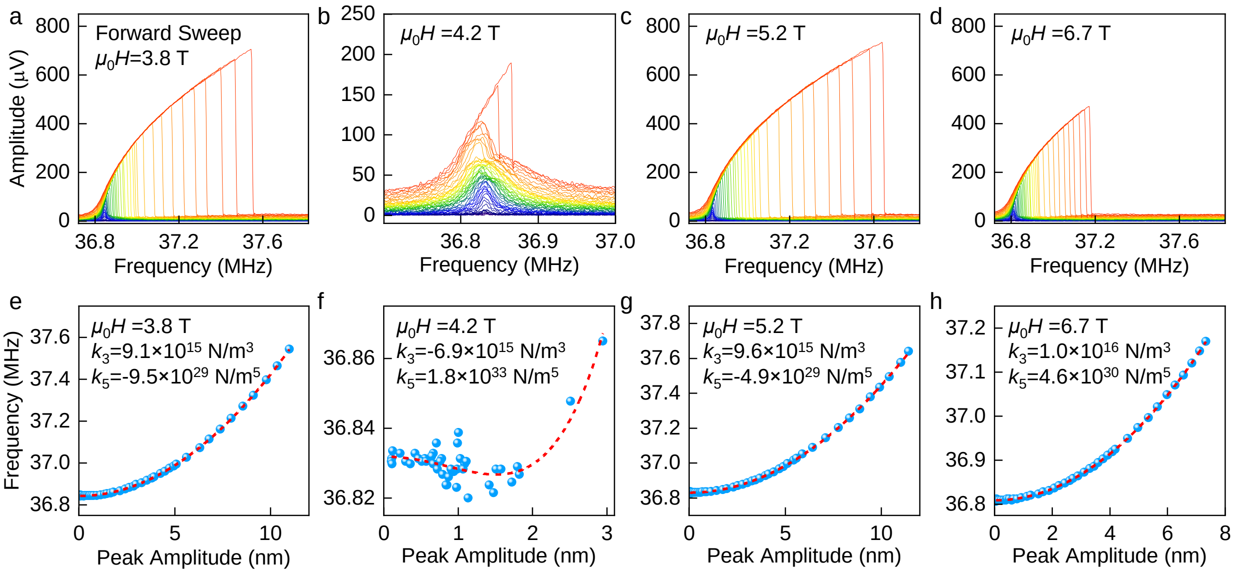

Figure S9| Nonlinear characterization of device 2 at 4.2 K at varying magnetic fields. Forward sweeps of the frequency response at **a,** $\mu_0 H$ =3.8 T, **b,** $\mu_0 H$ =4.1 T, **c,** $\mu_0 H$=5.2 T, and **d,** $\mu_0 H$ =6.7 T. **e-h**, Backbone curves corresponding to panel (a)-(d). Red dashed lines are fitted to Eq. S6.

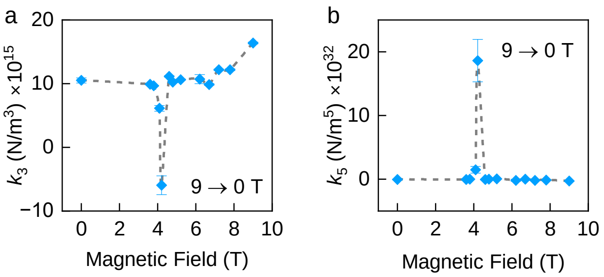

Figure S10| Nonlinear coefficient extraction during 9 T to 0 T magnetic field sweep. **a,** cubic (k_3) and **b,** quintic (k_5) nonlinear coefficient are extracted from the recorded forward sweeps at varying magnetic fields. The grey lines in both panels are added to guide the eyes.

S10 Additional Data on Nonlinear Characterization at Varying Magnetic Fields

We summarize the upper stable branch to lower stable branch transition frequency (f_{fd}) during forward frequency sweeps, lower stable branch to upper stable branch transition frequency (f_{bd}) during backward frequency sweeps, the corresponding amplitude and the difference between amplitudes in Fig. S11.

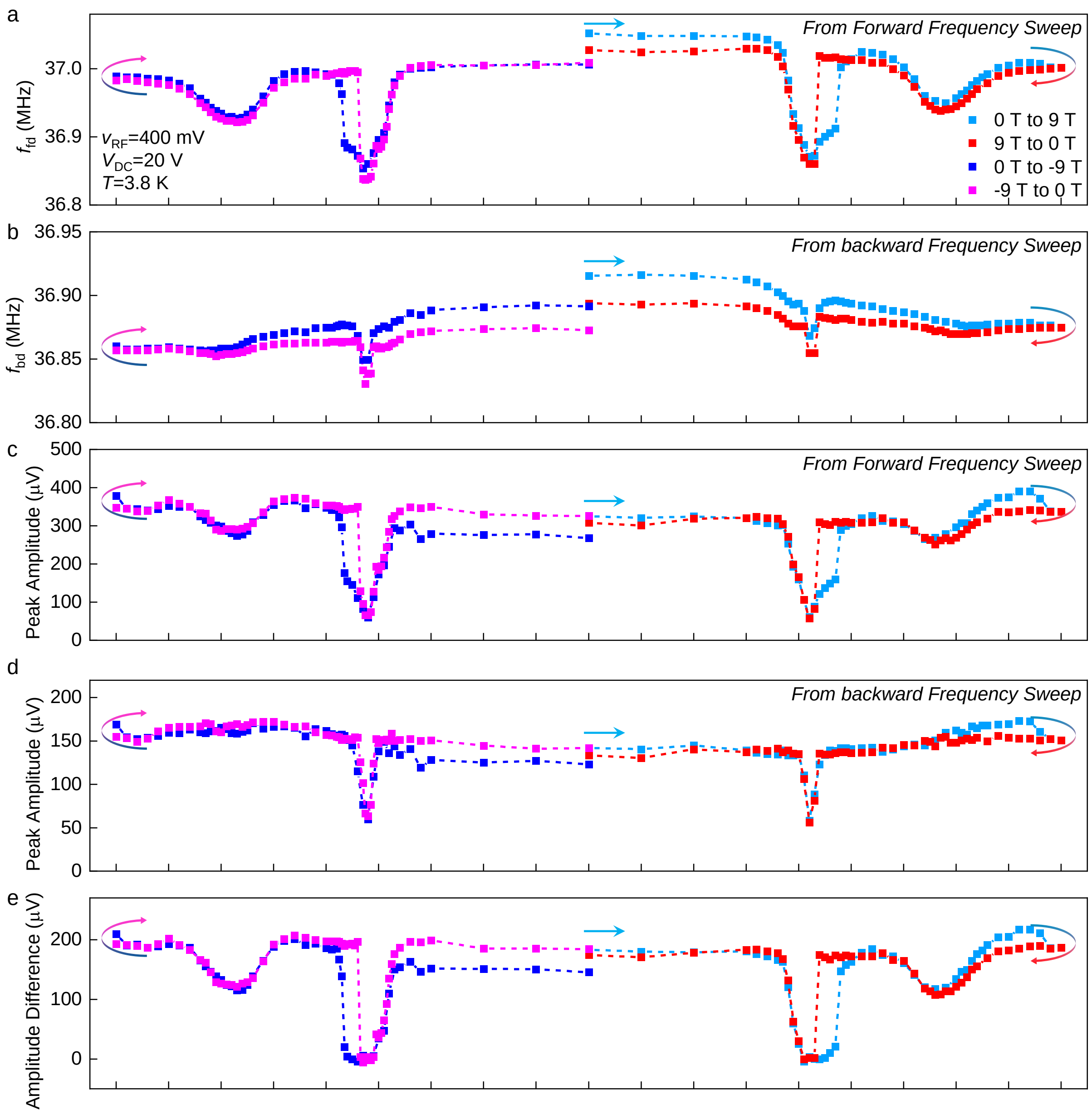

Figure S11| Nonlinear characterization at varying magnetic field. **a**, Upper stable branch to lower stable branch transition frequency (f_{fd}) during forward frequency sweeps from the network analyzer. **b**, Lower stable to upper stable branch transition frequency (f_{bd}) frequency during backward frequency sweeps. Upper stable branch amplitude corresponding to **c,** panel (**a**) and **d,** panel (**b**). **e,** Amplitude difference between panel (**d**) and (**e**) showing clear transition fields at both H_1 and H_2.

S11 Frequency Stability Analysis

The detection sensitivity of the sensor is ultimately determined by the frequency fluctuation noise in a resonance-frequency-shift-based detection technique. Hence, it is crucial to evaluate the frequency stability of the oscillator. Allan deviation (σ_A) defined by[8]

$$\sigma_A(\tau_A) = \left[\frac{1}{2(N-1)}\sum_{b=1}^{N-1}\left(\frac{\bar{f}_{b+1}-\bar{f}_b}{f_0}\right)^2\right]^{\frac{1}{2}} \quad (S7)$$

quantifies the frequency stability of the oscillator, where $\bar{f}_b$ is the averaged frequency in the bth time interval of τ_A and f_0 is the nominal carrier frequency. We measure the frequency fluctuation of device 2 operating at 4.2 K for 25 min (Fig. S12a) using a phase locked loop (PLL) from Zurich Instruments (UHFLI 600MHz Lock-in Amplifier). From the raw data, we estimate the Allan deviation using Eq. S7. The resonator shows short-term stability $\sigma_A \approx 6\times10^{-11}$ at τ_A=4 s and long-term stability $\sigma_A \approx 2\times10^{-10}$ at τ_A=400 s (Fig. S12b). The Allan deviation follows $\tau_A^{-1/2}$ power law (frequency stability limit imposed by thermomechanical motion) for short-term averaging (τ_A from 0.5 s to 4 s) and τ_A^1 power law (frequency stability limit imposed by drift) for long-term averaging (τ_A from 10 s to 400 s).

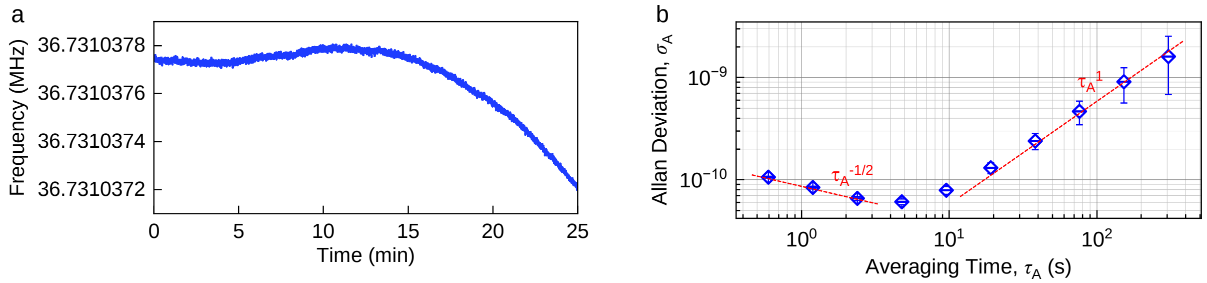

Figure S12| Frequency stability analysis. **a,** Recorded frequency of device 2 over 25 min. **b,** Computed Allan deviation using Eq. S7 showing $\tau_A^{-1/2}$ and τ_A^1 power law.

S12 Measured Resonance Response at 100 K

We performed the resonance characterization at varying magnetic fields above the Neel temperature of $MnPS_3$. We sweep between -14 T to 14 T and record the corresponding response. At this elevated temperature above the Neel temperature, the two sharp transitions are not observed (Fig. S13).

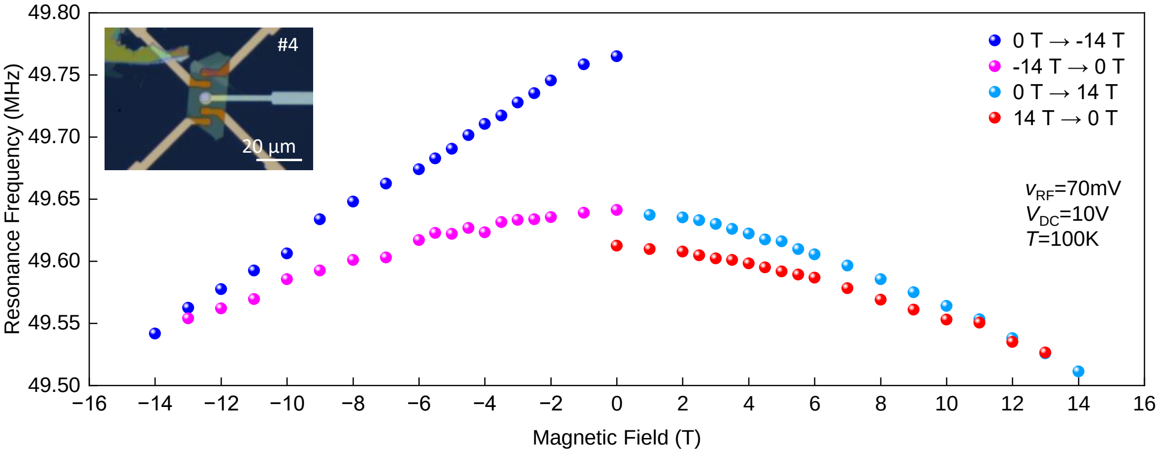

Figure S13| Measured resonance response above the Neel temperature of $MnPS_3$.

S13 Estimation of Strain Change

The resonance frequency of a drumhead resonator can be expressed as

$$f_{0,0\mathrm{T}} = \frac{2.4048}{2\pi r}\sqrt{\frac{\gamma_{0\mathrm{T}}}{\rho h}} = \frac{2.4048}{2\pi r}\sqrt{\frac{\varepsilon_{0\mathrm{T}} E_{\mathrm{Y,3D}}}{\rho}}$$

We can rewrite this equation to find strain of the 2D membrane as

$$\varepsilon_{0\mathrm{T}} = \frac{0.691675\pi^2 \rho r^2}{E_{\mathrm{Y,3D}}} f_{0,0\mathrm{T}}^2 \tag{S8}$$

By taking Taylor's expansion around a small change in resonance frequency (Δf_0), we obtain,

$$\varepsilon = \frac{0.691675\pi^2 \rho r^2}{E_{\mathrm{Y,3D}}} f_{0,0\mathrm{T}}^2 + 2 \times \frac{0.691675\pi^2 \rho r^2}{E_{\mathrm{Y,3D}}} f_{0,0\mathrm{T}}(\Delta f_0) = \varepsilon_{0\mathrm{T}} + \Delta\varepsilon \tag{S9}$$

From the expression of the change in strain ($\Delta\varepsilon$), we estimate a strain change of ~2×10^{-8} for 1 kHz shift in the resonance frequency ($E_{\mathrm{Y,3D}}$=770 GPa at 4 K, device 1).

S14 Summary of Measured Results

We summarize the measured results for different devices with varying diameter and thickness in Table S1. The measured value of H_1 and H_2 transitions increase with layer thickness.

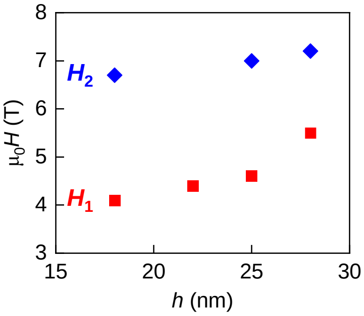

Figure S14| Measured H_1 and H_2 transitions at varying layer thickness.

Table S1: Summary of measured results at 4 K

Parameters	Device 1	Device 2	Device 3	Device 4
d (μm)	6	6	7	4
h (nm)	25	18	28	22
f_0 at 298 K (MHz)	11.60	10.85	16.38	23.80
f_0 at 4 K (MHz)	38.94	36.68	31.59	57.59
Q at 4 K	56,000	5,000	12,000	40,000
$\mu_0 H_1$ (T)	4.6	4.1	5.5	4.4
$\mu_0 H_2$ (T)	7	6.7	7.2	-

S15 Simulation Methods and Results

Atomistic simulations were performed using the VAMPIRE software [9] to calculate the magnetic properties of $MnPS_3$. The system was described using the spin Hamiltonian:

$$\mathcal{H} = -\frac{1}{2}\sum_{i\neq j} J_{ij}\, \boldsymbol{S_i}\cdot\boldsymbol{S}_j - \frac{1}{2}B \sum_{\mathrm{NN};i\neq j} \left(\boldsymbol{S}_i\cdot\boldsymbol{S}_j\right)^2 - K\sum_i (S_i^z)^2 \qquad \text{(S10)}$$

where $\boldsymbol{S_{i,j}}$ are unit vectors representing the local spin directions on sites i,j. The first term represents bilinear exchange with the exchange constant J_{ij} between spins i and j. The bilinear exchange was considered up to the third nearest neighbor for in-plane and out-of-plane neighbors. The second term describes the biquadratic exchange contribution between sites i and its in-plane nearest neighbors and B is the biquadratic exchange constant. Both the bilinear and biquadratic interactions are isotropic. The last term is the easy-axis anisotropy contribution with the magnetocrystalline constant K. Table S2 gives the exchange parameters used for the calculations.

Table S2: In-plane bilinear exchange constants $J_{1,2,3}$ up to third nearest neighbor, out-of-plane bilinear exchange constants $J_{4,5,6}$ up to third nearest neighbor, biquadratic exchange constant B and easy-axis anisotropy constant K for $MnPS_3$. In-plane exchange couplings J_1-J_3, B and K were taken from [10] and interlayer exchange couplings J_4-J_6 were taken from [11].

Parameter	Energy (meV)	Reference
J_1	-7.89	[10]
J_2	-0.21	[10]
J_3	-3.47	[10]
J_4	-0.269	[11]
J_5	-0.107	[11]
J_6	-0.0538	[11]
B	0.95	[10]
K	0.025	[10]

Spin-dynamics simulations were undertaken to determine the stability and motion of domain walls in $MnPS_3$ ribbons of dimensions 200 nm × 50 nm with 1-7 layers. The simulations were performed by solving the Landau-Lifshitz-Gilbert (LLG) equation:

$$\frac{\partial \boldsymbol{S}_i}{\partial t} = -\frac{\gamma_e}{1+\alpha^2}[\boldsymbol{S}_i \times \boldsymbol{B}_{\text{eff}} + \alpha \boldsymbol{S}_i \times (\boldsymbol{S}_i \times \boldsymbol{B}_{\text{eff}})] \quad \text{(S11)}$$

which models the interaction of an atomic spin moment $\boldsymbol{S}_i$ with an effective magnetic field $\boldsymbol{B}_{\text{eff}} = -1/\mu_s \frac{\partial \mathcal{H}}{\partial \boldsymbol{S}_i}$. A modified Heun integration method was utilized to increase simulation times up to several nanoseconds. A time step of 0.1 fs, a Gilbert damping parameter α =0.01 [12] and a magnetic moment μ_B =4.68 [10] for the Mn atoms were used in all simulations. To study domain wall motion, a domain wall was artificially created in the input files and stabilized by field cooling from 0.01 K to 0 K over 0.2 ns, and then the simulation was continued up to 0.4 ns. Then the external magnetic field was applied along the *z*-axis and the LLG equation was solved to study domain wall dynamics up to a simulation time of 2 ns. The spin dynamics simulations were carried out at 0 K to isolate the effect of the magnetic field on domain wall motion.

The domain wall velocity was extracted from the motion of the domain wall center of mass. The atomistic spin configuration of the $MnPS_3$ ribbon was stored at time intervals of 0.01 ns. At each time interval, the domain wall width and position of the center of mass were calculated by fitting the *z*-component of spin, m_z, with the function:

$$m_z(x) = \cos\left(2\arctan\left[\frac{x - x_0}{\Delta}\right]\right) \quad \text{(S12)}$$

where x_0 is the centre of mass of the domain wall and Δ is the Bloch parameter related to Domain Wall width [13,14]. The data was fit for the spin chain at the center of the ribbon to reduce edge effects on the fitting. At each interval, the values of x_0 and Δ were taken from the fit data of the previous interval. The domain wall velocity was then calculated by differentiating the position of the centre of mass with time. An example of the domain wall fitting for 5-layer $MnPS_3$ at 2 T is shown in Fig. S15.

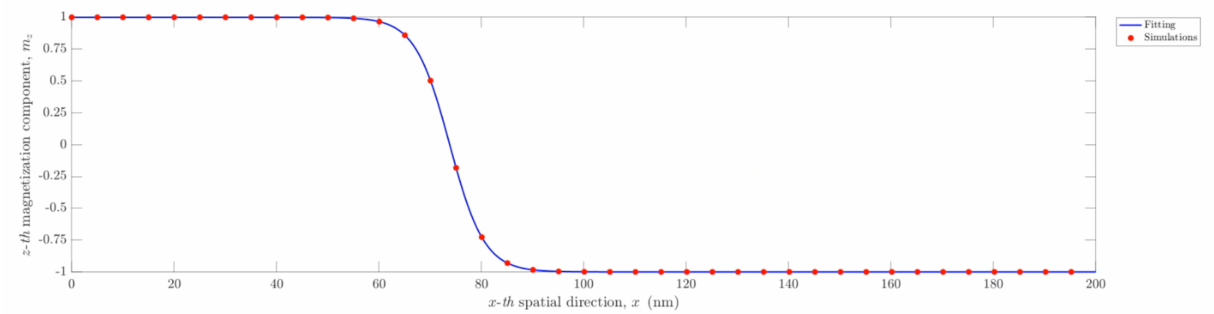

Figure S15| Domain wall fitting for 5-layer $MnPS_3$ with an external field of 2 T. The m_z data is fit to Eq. S12 for the middle spin-chain (y ~25 nm) of the $MnPS_3$ ribbon. The temperature is 0 K.

Below the spin-flop field, the domain wall fitting is straightforward as the spins are primarily aligned along z as shown in Fig. S18. However, close to the spin-flop field, there are spin waves emitted in x and y as shown in Fig. S19. This makes the fitting more difficult as the in-plane components of the spin start to dominate. Figure S16 shows the fitting for 5-layer $MnPS_3$ at a field of 7.4 T, close to the spin-flop field in our simulations. The fitting quality is slightly reduced as m_z no longer goes from 1 to -1 but is still accurate enough to extract the domain wall velocity. Beyond the spin-flop transition field, the in-plane components of spin dominate and Eq. S12 can no longer be used to fit the data as shown in Fig. S17.

We can see that spin waves are only emitted at and beyond the spin-flop transition field (see SI Movies). At 7.4 T we observe spin wave emission up till ~1 ns after the external field is applied. The spins finally reach a canted configuration between the x and z axes. However, at 8 T, the spin wave emission happens over ~0.3 ns and the final configuration is mostly in-plane along the x direction with slight out-of-plane canting. This could imply that the spin-flop transition is a continuous transition with dissipation that could cause the drop in the Q factor observed in Figure 2b.

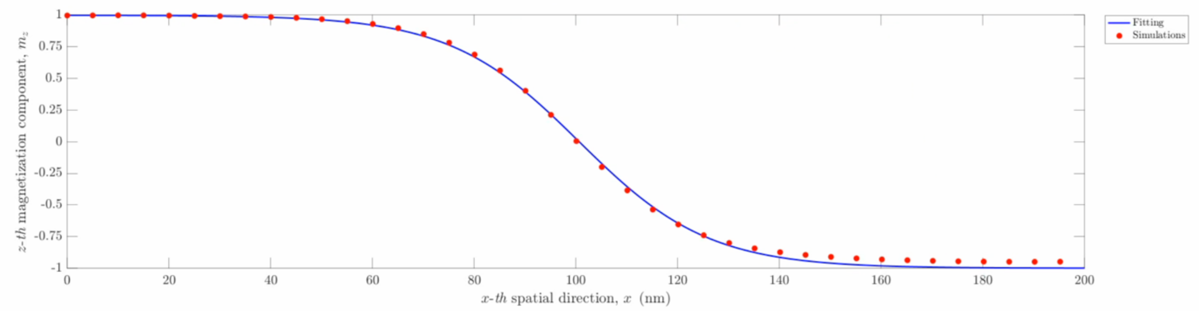

Figure S16| Domain wall fitting for 5-layer $MnPS_3$ with an external field of 7.4 T. The m_z data is fit to Eq. S12 for the middle spin-chain (y ~25 nm) of the $MnPS_3$ ribbon. The temperature is 0 K.

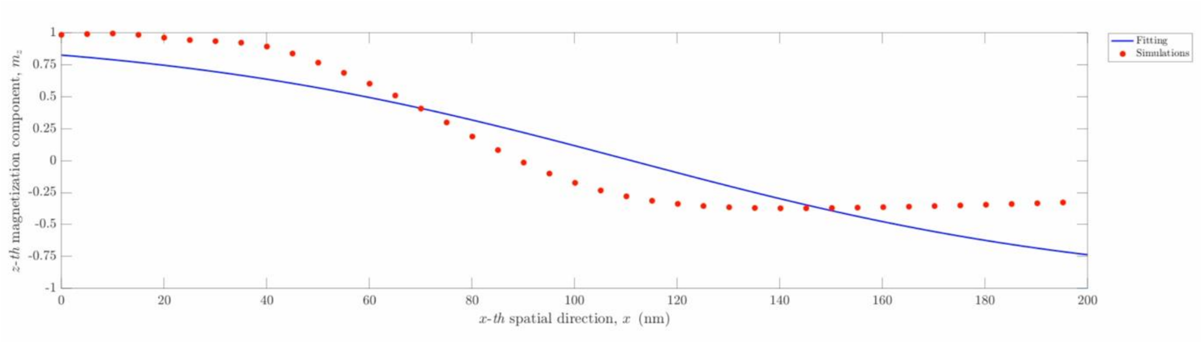

Figure S17| Domain wall fitting for 5-layer $MnPS_3$ with an external field of 8 T. The m_z data is fit to Eq. S12 for the middle spin-chain (y ~25 nm) of the $MnPS_3$ ribbon. The temperature is 0 K.

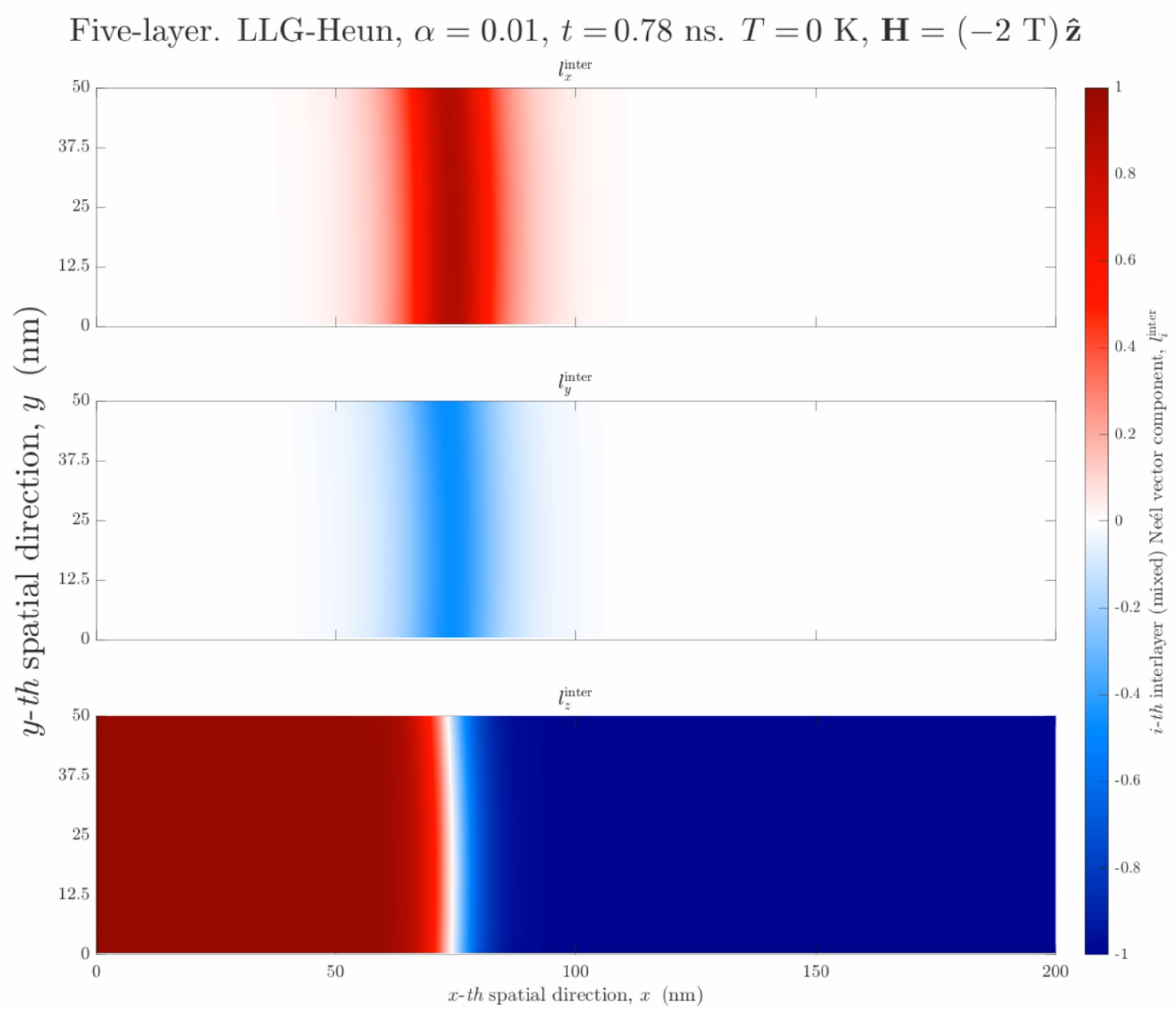

Figure S18| x, y and z components of spin of the $MnPS_3$ ribbon at a field of 2 T. The snapshot is taken at the same time step as the fitting data in Fig. S15.

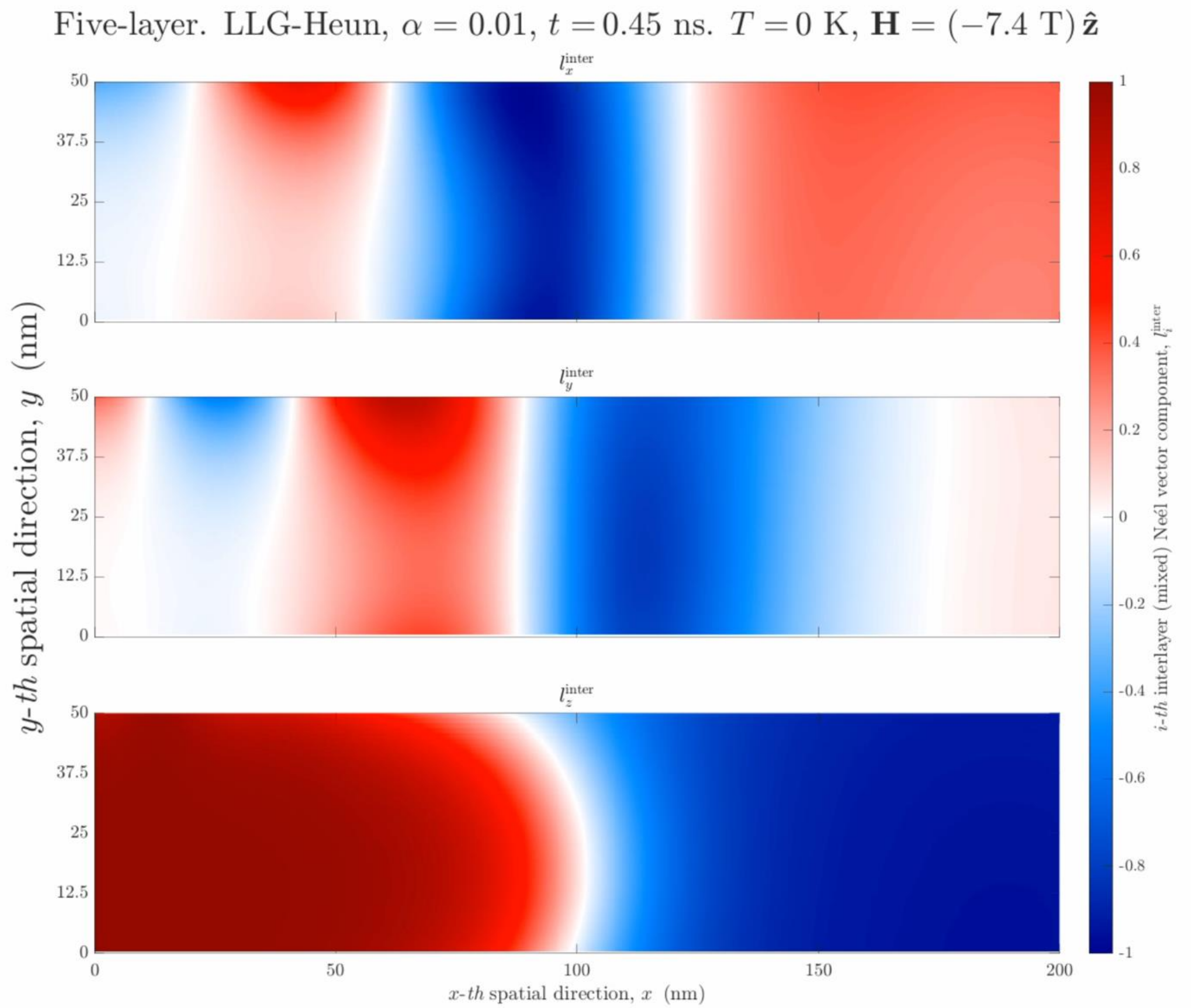

Figure S19| x, y and z components of spin of the $MnPS_3$ ribbon at a field of 7.4 T. The snapshot is taken at the same time step as the fitting data in Fig. S16 to highlight the difficulty in fitting the data at higher fields.

The videos with the x, y and z components of spin for the $MnPS_3$ ribbon for 1, 3, 5 and 7 layers at fields 2 T, 7 T, 7.4 T and 8 T are available at this link.

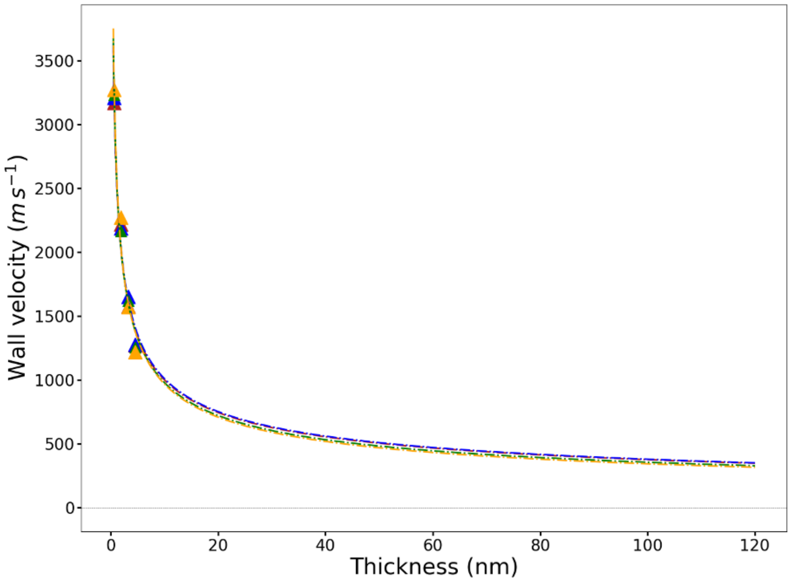

Figure S20| Extrapolation of DW velocity near the spin-flop transition fields as a function of layer thicknesses in the simulation. The scattered data of different colors represent the simulated DW speeds within (1±5%) H_{SF} field window. The solid lines are the power law fitting to extrapolate to the bulk limit.

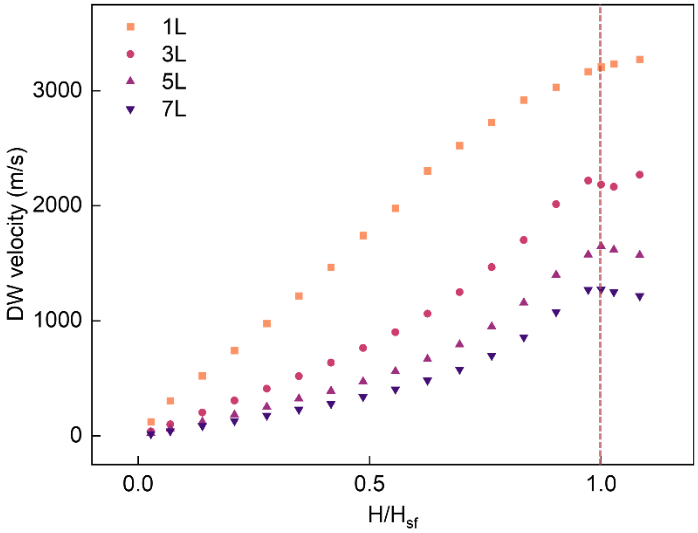

Figure S21| Simulated domain wall velocity for different layers, which show a maxima at the H_{sf} as denoted by the red dashed line.

Data Availability

The input files for the VAMPIRE simulations for the various layers and fields are available at the repository: https://github.com/shreyas-ramachandran/mnps3_domain_wall_motion

References

1. S M E. H. Yousuf, J. Lee, S. W. Shaw, and P. X.-L. Feng, “Phononic frequency combs in atomically thin nanoelectromechanical resonators via 1:1 and 2:1 internal resonances”, *J. Microelectromech. Syst.*, vol. 32, no. 4, pp. 335-346 (2023).

2. H. Suzuki, N. Yamaguchi, and H. Izumi, “Theoretical and experimental studies on the resonance frequencies of a stretched circular plate: Application to Japanese drum diaphragms”, *Acoust. Sci. Technol.*, vol. 30, pp. 348–354 (2009).

3. Šiškins, M. et al., “Magnetic and electronic phase transitions probed by nanomechanical resonators”, *Nat. Commun.*, vol. 11, art. no. 2698 (2020).

4. Pierre Villars (Chief Editor), PAULING FILE in: Inorganic Solid Phases, SpringerMaterials (online database), Springer, Heidelberg (ed.) SpringerMaterials.

5. J. Zhu, B. Xu, F. Xiao, Y. Liang, C. Jiao, J. Li, Q. Deng, S. Wu, T. Wen, S. Pei, J. Xia, and Z. Wang, “Frequency scaling, elastic transition, and broad-range frequency tuning in WSe_2 nanomechanical resonators”, *Nano Lett.*, vol. 22, no. 13, pp. 5107-5113 (2022).

6. J. Lee, Z. Wang, K. He, R. Yang, J. Shan, and P. X.-L. Feng, “Electrically tunable single- and few-layer MoS_2 nanoelectromechanical systems with broad dynamic range,” *Sci. Adv.*, vol. 4, no. 3, art. no. eaao665 (2018).

7. T. Kaisar, J. Lee, D. Li, S. W. Shaw, and P. X.-L. Feng, “Nonlinear stiffness and nonlinear damping in atomically thin MoS_2 nanomechanical resonators,” *Nano Lett.*, vol. 22, no. 24, pp. 9831–9838 (2022).

8. T. Kaisar, S M E. H. Yousuf, J. Lee, A. Qamar, M. Rais-Zadeh, S. Mandal, and P. X.-L. Feng, “Five low-noise stable oscillators referenced to the same multimode AlN/Si MEMS resonator”, *IEEE Trans. Ultrason., Ferroelectr., Freq. Control*, vol. 70, no. 10, pp. 1213-1228 (2023).

9. R. F. L. Evans, W. J. Fan, P. Chureemart, T. A. Ostler, M. O. A. Ellis, and R. W. Chantrell, “Atomistic spin model simulations of magnetic nanomaterials”, *J. Phys.: Condens. Matter.*, vol. 26, no. 10, art. no. 103202 (2014).

10. M. Amirabbasi and P. Kratzer, “Effect of biquadratic magnetic exchange interaction in the 2D antiferromagnets $M\,PS_3$ (M = Mn,Fe,Co,Ni)”, *Phys. Rev. Mater.*, vol. 8, no. 8, art. no. 084005 (2024).

11. D. Vaclavkova, A. Delhomme, C. Faugeras, M. Potemski, A. Bogucki, J. Suffczyński, P. Kossacki, A. R. Wildes, B. Grémaud, and A. Saúl, “Magnetoelastic interaction in the two-

dimensional magnetic material $MnPS_3$ studied by first principles calculations and Raman experiments", *2D Mater.*, vol. 7, no. 3, art. no. 035030 (2020).

12. I. M. Alliati, R. F. L. Evans, K. S. Novoselov, and E. J. G. Santos, "Relativistic domain-wall dynamics in van der Waals antiferromagnet $MnPS_3$", *npj Comput. Mater.*, vol. 8, art. no. 3 (2022).

13. N. L. Schryer and L. R. Walker, "The motion of 180° domain walls in uniform dc magnetic fields", *J. Appl. Phys.*, vol. 45, no. 12, pp. 5406–5421 (1974).

14. R. Rama-Eiroa, P. E. Roy, J. M. González, K. Y. Guslienko, J. Wunderlich, and R. M. Otxoa, "Inertial domain wall characterization in layered multisublattice antiferromagnets", *J. Magn. Magn. Mater.*, vol. 560, art. no. 169566 (2022).
